# Multifunctional Boron‐based 2D Nanoplatforms Ameliorate Severe Respiratory Inflammation by Targeting Multiple Inflammatory Mediators

**DOI:** 10.1002/advs.202412626

**Published:** 2025-02-14

**Authors:** Changyi Xu, Ming Liu, Xinran Xie, Zhixin Li, Yuefei Zhu, Yang Ye, Mengya Du, Suhua Hu, Tianrun Liu, Yubiao Guo, Weiping Wen, Huanliang Liu, Zhaoxu Tu

**Affiliations:** ^1^ Department of Otolaryngology The Sixth Affiliated Hospital Sun Yat‐sen University Guangzhou Guangdong 510655 China; ^2^ Department of Clinical Laboratory The Sixth Affiliated Hospital Sun Yat‐sen University Guangzhou Guangdong 510655 China; ^3^ Biomedical Innovation Center The Sixth Affiliated Hospital Sun Yat‐sen University Guangzhou Guangdong 510655 China; ^4^ Department of Biomedical Engineering Columbia University New York 10027 USA; ^5^ Department of Pulmonary and Critical Care Medicine The First Affiliated Hospital Sun Yat‐Sen University Guangzhou Guangdong 510655 China; ^6^ Department of Otolaryngology The First Affiliated Hospital Sun Yat‐sen University Guangzhou Guangdong 510655 China

**Keywords:** bacterial infection, boron‐based nanosheets, neutrophil extracellular traps, reactive oxygen and nitric species, respiratory inflammation

## Abstract

Effective management of serious respiratory diseases, such as asthma and recalcitrant rhinitis, remains a global challenge. Here, it is shown that induced sputum supernatants (ISS) from patients with asthma contain higher levels of cell‐free DNA (cfDNA) compared to that of healthy volunteers. Although cfDNA scavenging strategies have been developed for inflammation modulation in previous studies, this fall short in clinical settings due to the excessive neutrophil extracellular trap (NET) formation, reactive oxygen and nitrogen species (RONS) and bacterial infections in injured airway tissues. Based on this, a multifunctional boron‐based 2D nanoplatform B‐P_M_ is designed by coating boron nanosheets (B‐NS) with polyamidoamine generation 1 (PG1) dendrimer, which can simultaneously target cfDNA, NETs, RONS, and bacteria. The effects of B‐P_M_ in promoting mucosal repair, reducing airway inflammation, and mucus production have been demonstrated in model mice, and the therapeutic effect is superior to dexamethasone. Furthermore, flow cytometry with clustering analysis and transcriptome analysis with RNA‐sequencing are adopted to comprehensively evaluate the in vivo anti‐inflammation therapeutic effects. These findings emphasize the significance of a multi‐targeting strategy to modulate dysregulated inflammation and highlight multifunctional boron‐based 2D nanoplatforms for the amelioration of respiratory inflammatory diseases.

## Graphical abstract

There are many limitations of conventional corticosteroids in the treatment of respiratory inflammation. Here, a multifunctional 2D nanoplatform is designed that simultaneously targets cfDNA, NETs, RONS, and bacterial infection. The nanoplatforms exhibited excellent therapeutic efficacy in vivo and could serve as a new therapeutic approach for severe respiratory inflammation.

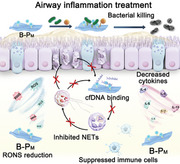

## 1 Introduction

Recalcitrant respiratory disorders, including asthma, chronic obstructive pulmonary disease (COPD), chronic rhinosinusitis with nasal polyps (CRSwNP), and others, affect millions of people globally.^[^ ^1^, ^2^, ^3^ ^]^ Alarmingly, the prevalence of these conditions continues to rise, placing a significant burden on healthcare systems and society.^[^ ^4^ ^]^ The commonly used clinical drugs (such as glucocorticoids) are largely limited by their lack of effectiveness and significant side effects after treatment.^[^ ^5^ ^]^ Therefore, it is crucial to develop new therapeutic strategies that can minimize systemic side effects while achieving satisfactory treatment effects.

Neutrophil infiltration in airway tissues has been reported to play a pivotal role in many patients with severe respiratory disorders,^[^ ^6^, ^7^ ^]^ and this process is closely associated with glucocorticoid resistance.^[^ ^8^ ^]^ Neutrophils, as the first line of immune defense, can form neutrophil extracellular traps (NETs) composed of dsDNA, citrullinated histone H3 (CitH3), and granule proteins such as myeloperoxidase (MPO) in response to specific stimuli, such as bacterial infection.^[^ ^9^, ^10^ ^]^ However, recent studies have shown elevated NET levels in the airways of patients with chronic respiratory diseases, and NET formation is closely related to persistent inflammatory response.^[^ ^9^ ^]^ Along with NETs, cell‐free DNA (cfDNA), which belongs to damage‐associated molecular patterns (DAMPs), was also found to be elevated at sites of inflammation.^[^ ^11^, ^12^, ^13^ ^]^ Initially, cfDNA released from damaged airway epithelium stimulates NET formation via toll‐like receptor 9 (TLR9) activation. Simultaneously, NET accumulation exacerbates epithelial injury, leading to increased cfDNA generation and more NET formation with dsDNA embedded, which together amplifies the inflammatory response. Additionally, NET formation promotes immune cell infiltration and mucus production in the airway, while treatment with deoxyribonuclease I (DNase I) can effectively mitigate these effects by degrading cfDNA along with NETs.^[^ ^14^, ^15^ ^]^ These results indicate the critical role of cfDNA and NETs in respiratory disorders. However, the clinical application of DNase I has been largely restricted due to the significant side effects, high cost, and low stability.^[^ ^16^ ^]^

Lately, advances in nanomedicine have provided novel approaches for clinical diagnosis and treatment.^[^ ^17^, ^18^, ^19^ ^]^ Various animal models have validated that rationally engineered nanomaterials present a promising therapeutic approach for inflammation‐related diseases by efficiently eliminating excessive cfDNA and NETs.^[^ ^13^, ^20^, ^21^, ^22^, ^23^ ^]^ However, the therapeutic outcomes of nanomedicine for cfDNA scavenging remain suboptimal due to persistent challenges, such as elevated reactive oxygen and nitrogen species (RONS) and bacterial infection in inflamed airway tissues. Therefore, developing multifunctional nanoplatforms that can simultaneously address multiple inflammatory mediators in pathological tissues is crucial for the effective treatment of airway inflammation. As a well‐established biomedical polymer, polyamidoamine (PAMAM) has been widely applied in anti‐inflammation nanoplatforms.^[^ ^22^, ^24^, ^25^ ^]^ Due to its abundant surficial amine groups, PAMAM carries a robust positive charge, enabling it to bind to negatively charged nucleic acids (NAs) via electrostatic interaction, thereby suppressing the cfDNA/NETs‐induced inflammatory pathways.^[^ ^26^ ^]^ A recent study by Bu demonstrated that boron nanosheets (B‐NS), synthesized via the reaction of magnesium boride with water, exhibit both strong antibacterial properties, antioxidant capacity, and excellent biocompatibility.^[^ ^27^ ^]^ As a result, the fabrication of PAMAM generation 1 (PG1) modified B‐NS nanosheets (B‐P) is promising to develop novel multifunctional 2D nanoplatforms targeting multiple inflammatory mediators, including cfDNA and NETs elimination, antibacterial and antioxidant capabilities. In addition, nanoplatforms with 2D geometry and optimal size (100–500 nm) are less susceptible to macrophage phagocytosis and can be more effectively accumulated in the inflammatory tissues compared with spherical particles,^[^ ^28^, ^29^ ^]^ which is relatively beneficial for anti‐inflammation nanomedicine development.

In this work, we observed a significant increase in cfDNA levels in asthma patients' induced sputum supernatant (ISS), with cfDNA levels showing a negative correlation with pulmonary functions and a positive correlation with inflammation severity. This highlights the critical role of cfDNA in airway inflammation. Subsequently, we synthesized a series of B‐P nanosheets with varying sizes, and the nanosheets with a medium size (B‐P_M_) were selected with robust cfDNA binding, RONS reduction, anti‐bacterial properties, and favorable biocompatibility. Following this, the cfDNA and NETs elimination, antioxidant capacity, and antibacterial activity of B‐P_M_ were carefully studied in vitro. Subsequently, a mouse model with airway inflammation was established, and the biodistribution, biosafety, and anti‐inflammation effects of B‐P_M_ were investigated. We studied if B‐P_M_ treatment alleviates epithelial injury and mucus production in the airway of model mice and explored the potential mechanism through multi‐channel flow cytometry and RNA‐sequencing analysis. Finally, we meticulously examine the long‐term in vivo biosafety of the developed boron‐based nanosheets, elucidating their potential for clinical respiratory disorders therapy.

## 2 Results

### 2.1 The cfDNA Level in ISS and Its Correlation with Airway Symptoms

**Figure 1:**
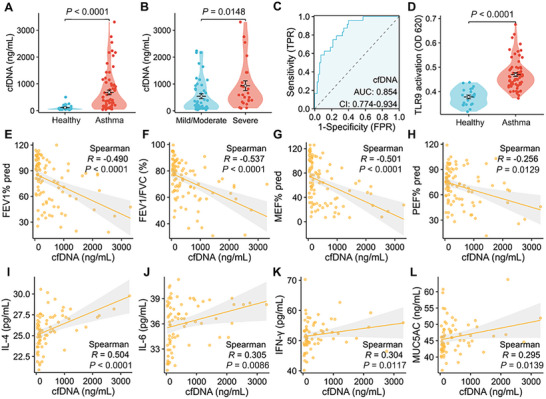
Analysis of cfDNA levels in ISS and its correlation with pulmonary function and airway inflammation in asthma patients. A) cfDNA levels in ISS of healthy volunteers and asthma patients. Data are expressed as mean ± SEM (Control: n = 24; Asthma: n = 70. Wilcoxon rank sum test, two‐tailed). B) cfDNA levels in ISS of mild/moderate and severe asthma patients. Data are expressed as mean ± SEM (Mild/moderate: n = 46; Severe: n = 24. Wilcoxon rank sum test, two‐tailed). C) ROC curve based on cfDNA level. TPR: true positive rate; FPR: false positive rate; AUC: area under curve; CI: confidence interval. D) TLR9 activation of HEK‐TLR9 cells induced by ISS of healthy volunteers and asthma patients. Data are expressed as mean ± SEM (Control: n = 21; Asthma: n = 67. Wilcoxon rank sum test, two‐tailed). E–L) Correlation of cfDNA level in ISS with FEV1% pred (n = 94), FEV1/FVC (%) (n = 94), MEF% pred (n = 92), PEF% pred (n = 94), and IL‐4 (n = 70), IL‐6 (n = 73), IFN‐γ (n = 69), and MUC5AC (n = 69) in ISS (Spearman correlation analysis). FEV1% pred: forced expiratory volume at 1 s to predicted value ratio, FVC: forced vital capacity, MEF% pred: maximum expiratory flow to predicted value ratio, PEF% pred: peak expiratory flow to predicted value ratio.

Although cfDNA was reported to be elevated in nasal secretions of several upper airway inflammations, such as CRSwNP,^[^ ^30^, ^31^ ^]^ the significance of cfDNA in lower airway disorders, such as asthma, has not been thoroughly analyzed. In this work, 70 asthma patients and 24 healthy volunteers (participant demographics provided in Table S1, Supporting Information) were included, and their induced sputum (IS) was collected. Subsequently, the supernatant of IS (ISS) was prepared by filtration and centrifugation after treatment with dithiothreitol (DTT). The results of the pico‐green assay demonstrated a significant elevation of cfDNA level in the ISS of asthma patients compared to healthy volunteers (679.2 ± 88.7 vs 112.4 ± 21.3 ng mL^−1^) (**Figure** **1**A). Furthermore, severe asthma patients displayed higher cfDNA levels (≈1.72 fold) than mild/moderate patients, indicating that cfDNA levels are highly related to the inflammatory severity of asthma patients (Figure 1B). The receiver operating characteristic (ROC) curve was constructed based on cfDNA levels, yielding an area under the curve of 0.854 (Figure 1C), highlighting the significance of cfDNA for asthma diagnosis. Based on previous studies, cfDNA could stimulate several inflammatory pathways, including NET formation through TLR9 activation. Here, we co‐cultured the ISS with HEK‐Blue hTLR9 cells and observed a more pronounced activation of TLR9 in cells cultured with ISS from asthma patients (Figure 1D). In addition, ISS from asthma patients also had an activation effect on TLR3, TLR7, and TLR8, which play crucial roles in immune regulation (Figure S1A–C, Supporting Information). Previous studies have indicated an elevation of the NET level in the airway mucosa of asthma patients,^[^ ^32^ ^]^ and NETs were reported to be involved in many inflammatory diseases. These results strongly indicate that cfDNA is involved in the pathogenesis of respiratory disorders and may contribute to the uncontrolled airway inflammatory response in asthma patients. In the next step, comprehensive correlation analyses were performed to elucidate the relationship between cfDNA levels and the inflammatory status of asthma patients. The results revealed that cfDNA levels were negatively correlated with FEV1% pred, FEV1/FVC%, MEF% pred, PEF% pred, MEF25% pred, MEF50% pred, and MEF75% pred (Figure 1E–H; Figure S2A–D, Supporting Information). In contrast, a positive correlation was observed between cfDNA levels and leukocyte and neutrophil counts in peripheral blood (Figure S2E,F, Supporting Information) as well as certain pro‐inflammatory cytokines (IL‐4, IL‐6, IFN‐γ, CLCA1, IL‐5, IL‐25, IL‐33, POSTN, TSLP, SERPINB2, IL‐2) in ISS (Figure 1I–K; Figure S2H–O, Supporting Information), along with a positive correlation with MUC5AC (Figure 1L). These findings collectively suggested that higher cfDNA concentrations were associated with poorer pulmonary function and dysregulated inflammation, indicating that cfDNA is a probable therapeutic target for asthma. Although DNase I, which could degrade cfDNA, was approved by the Food and Drug Administration (FDA) as a medicine for cystic fibrosis (CF) patients to improve pulmonary function, its clinical application is severely limited by unfavorable therapeutic effects, high costs, low stability, and side effects, such as voice changes and rash.^[^ ^33^ ^]^ Fortunately, the biomaterials‐based anti‐inflammation strategies developed in recent years showed promise for alleviating the severe inflammatory response of patients with airway disorders.

### 2.2 Preparation and Characterization of Multifunctional Boron Nanosheets

**Figure 2:**
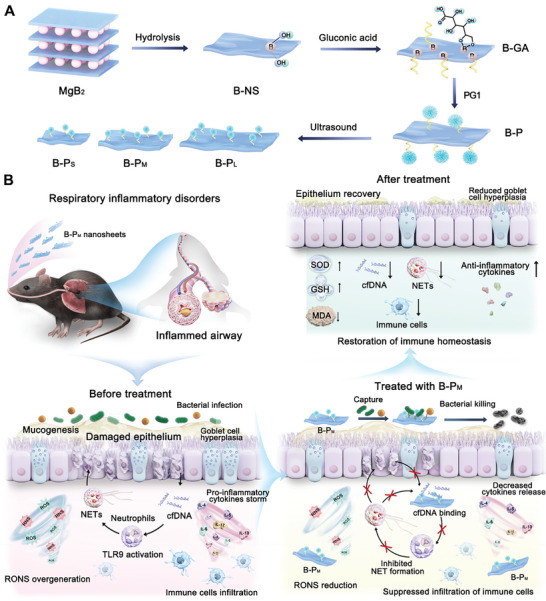
A schematic overview of B‐P_M_ construction and the alleviation of airway inflammation. A) The synthesis routes of B‐P_L_, B‐P_M_, and B‐P_S_. B) B‐P_M_ reduced inflammatory cell infiltration and mucus production via cfDNA/NETs scavenging, RONS reduction, and antibacterial effects.

**Figure 3:**
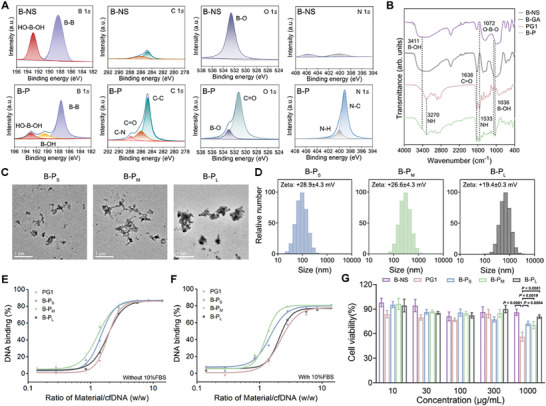
Characterizations of B‐NS, B‐P_S_, B‐P_M_, and B‐P_L_. A) XPS of B‐NS and B‐P. B) FTIR of B‐NS, B‐Gluconic acid (B‐GA), PG1 and B‐P. C) Representative TEM images of B‐P_S_, B‐P_M_, and B‐P_L_. Scale bars: 1 µm. D) Size and zeta potential of B‐P_S_, B‐P_M_ and B‐P_L_. E, F) cfDNA binding efficiency of PG1, B‐P_S_, B‐P_M_, and B‐P_L_ in PBS and PBS with 10%FBS. Data are presented as mean ± SD (n = 3). G) Viability of BEAS‐2B cells treated for 48 h with PG1, B‐P_S_, B‐P_M_, and B‐P_L_ with a series of concentrations. Data are presented as mean ± SD (n = 5).

In consideration of the significance of cfDNA, RONS, and bacterial infection in damaged airway tissues, a multi‐target combination therapeutic strategy is promising to attenuate the dysregulated inflammation (**Figure** **2**). Based on our previous studies, polyamidoamine generation 1 (PG1) was selected due to its robust cfDNA binding capacity and excellent biosafety. In addition, the conjugation of PG1 onto nanosheets to construct functional 2D nanoplatforms could further improve the cfDNA binding efficacy and the accumulation in the inflammatory tissues. Recently, boron‐based nanosheets (B‐NS), as newly emerged 2D nanomaterials obtained through the hydrolysis of MgB_2_ (**Figure** **3**A; Figure S3, Supporting Information), exhibited promising antibacterial and antioxidant effects.^[^ ^27^ ^]^ Based on these properties, the modification of PG1 onto the surface of B‐NS is promising to prepare the multifunctional 2D nanoplatforms targeting multiple inflammatory mediators. In detail, gluconic acid (GA) was attached to B‐NS by an esterification reaction between 1,3‐diol with boron dihydroxyl groups, and then PG1 was conjugated by an amidation reaction. X‐ray photoelectronic spectroscopy (XPS) revealed a decrease in the content of HO‐B‐OH in B‐P compared to B‐NS, along with the appearance of characteristic peaks such as C═O and C─N in the chemical structure of PG1 (Figure 3A; Figure S5, Supporting Information). Fourier‐transform infrared spectroscopy (FTIR) also confirmed the specific peaks of C═O and N─H bonds appeared after the modification of PG1 onto nanosheets (Figure 3B), indicating that B‐P was successfully prepared. Finally, B‐P with small size (B‐P_S_, 80–100 nm), medium size (B‐P_M_, 200–300 nm), and large size (B‐P_L_, 500–700 nm) were obtained by ultrasound (500 W) for 30, 15, and 5 min, respectively. The functionalized nanosheets with different sizes were also confirmed by transmission electron microscopy (TEM) (Figure 3C), and their Zeta potentials were +28.9 ± 4.3, +26.6 ± 4.3, and +19.4 ± 0.3 mV, respectively (Figure 3D; Figure S6, Supporting Information).

Next, we examined the cfDNA binding capacity of B‐P_S_, B‐P_M_, and B‐P_L_ with a series of concentrations (weight ratio of Material/cfDNA (w/w) from 0.14 to 14). The results demonstrated that B‐P_M_ exhibited significantly superior cfDNA scavenging capacity compared to B‐P_S_ and B‐P_L_ when the materials/cfDNA ratio was ≈2 (Figure 3E). This may be attributed to that B‐P_M_ displayed a larger size than B‐P_S_, allowing for greater adsorption and wrapping effect of cfDNA. While B‐P_M_ demonstrated a higher zeta potential than B‐P_L_, resulting in stronger electrostatic interaction with negatively charged cfDNA. These results collectively demonstrated the cfDNA binding capacity of functionalized boron nanosheets was controlled by the combination of size and surface charge. Similar results were observed in the medium supplemented with 10% fetal bovine serum (FBS) (Figure 3F), which was owing to the slight protein adsorption capacity (less than 7%) of functionalized nanosheets (Figure S7A, Supporting Information). Cell counting kit‐8 (CCK8) assay was adopted to evaluate the cell viability of human bronchial epithelial cells (BEAS‐2B) after 48 h incubation with nanosheets. The results revealed that B‐Ps, B‐P_M_, and B‐P_L_ exhibited excellent biosafety even at concentrations as high as 1000 µg mL^−1^, with cell viability over 70% (Figure 3G). In addition, the hemolysis test demonstrated that increasing concentrations of B‐P_M_ did not result in hemolysis of human peripheral blood even at 1000 µg mL^−1^ (Figure S7B, Supporting Information). More interestingly, B‐P_M_ remained relatively stable for at least 30 days at 4 °C, which is significant for the storage and transportation of nanomedicine in future applications (Figure S7C,D, Supporting Information). In addition, the size and zeta potential of B‐P_M_ in PBS did not change obviously after 5 days of incubation, confirming the stability for in vivo administration (Figure S7E,F, Supporting Information). According to the above results, B‐P_M_ with optimal cfDNA scavenging capacity and excellent biocompatibility was selected for subsequent experiments.

### 2.3 B‐P_M_ Inhibited TLR9 Activation and NET Formation

**Figure 4:**
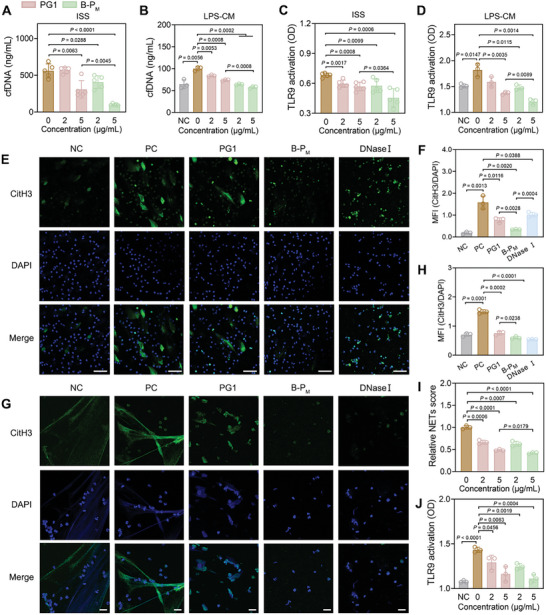
B‐P_M_ suppressed cfDNA‐induced TLR9 activation and NET formation. A,B) cfDNA in ISS (n = 5) and LPS‐CM (n = 3) after incubation with PG1 and B‐P_M_. C,D) TLR9 activation of HEK‐TLR9 cells induced by ISS (n = 5) and LPS‐CM (n = 3) after incubation with PG1 and B‐P_M_. E) Representative DAPI and CitH3 co‐staining images of the LPS‐CM‐treated neutrophils after incubation with PG1, B‐P_M_, and DNase I. Scale bars: 50 µm. F) Quantitative analysis of the ratio of CitH3/DAPI in (E) (n = 3). G) Representative DAPI and CitH3 co‐staining images of the NETs produced by LPS‐treated neutrophils after incubation with PG1, B‐P_M_, and DNase I. Scale bars: 50 µm. H) Quantitative analysis of the ratio of CitH3/DAPI in (G) (n = 3). I) The relative content of NETs after incubation with PG1 and B‐P_M_ (n = 3). J) TLR9 activation of HEK‐TLR9 cells induced by NETs after incubation with PG1 and B‐P_M_ (n = 3). Neutrophils incubated with medium‐only were negative control (NC) and neutrophils incubated with LPS or LPS‐CM were positive control (PC). Data are presented as mean ± SD (Student's t‐test, two‐tailed).

The clinical data demonstrated an elevation of cfDNA levels in the ISS of patients with asthma, and then we explored the application of B‐P_M_ as multiple‐targeting nanoplatforms for airway inflammation treatment. BEAS‐2B cells were treated with lipopolysaccharide (LPS), and a significant elevation of cfDNA levels was detected in the LPS‐conditioned medium (LPS‐CM) (Figure S8, Supporting Information). Subsequent treatment with B‐P_M_ resulted in a decrease in cfDNA content in both ISS and LPS‐CM. Notably, the cfDNA scavenging effect of B‐P_M_ was superior to that of PG1; B‐P_M_ (5 µg mL^−1^) can reduce the cfDNA in ISS from 560.4 ± 95.13 to 100.0 ± 12.82 ng mL^−1^, while the value in PG1 group is 309.5 ± 119.4 ng mL^−1^, confirming the advantage of the 2D nanostructure for cfDNA binding (**Figure** **4**A,B).

As cfDNA was reported to induce excessive inflammation via TLR9 activation, such as degranulation of neutrophils and NET formation, we investigated whether B‐P_M_ could suppress cfDNA‐induced TLR9 activation. Our findings revealed that both ISS and LPS‐CM were capable of activating TLR9, and the addition of B‐P_M_ significantly reduced TLR9 activation. More importantly, the suppression of TLR9 was more efficient for B‐P_M_ in comparison to the PG1‐treated group at 5 µg mL^−1^, confirming the superiority of the planar backbone for anti‐inflammation nanomedicine (Figure 4C,D). In addition, a similar inhibitory effect was observed for TLR3 (Figure S9A,B, Supporting Information).

In the next step, we examined the influence of B‐P_M_ on NET formation by stimulating neutrophils with LPS‐CM. The results showed that LPS‐CM induced the degranulation of neutrophils followed by NET release, while NET formation was suppressed by B‐P_M_ and PG1 treatment, and the mean fluorescence intensity (MFI) of CitH3/DAPI decreased to 21.5% and 48.8%, respectively (Figure 4E,F). These results suggested that B‐P_M_ can inhibit NET formation by scavenging cfDNA. Previous studies have reported that DNase I can degrade cfDNA and also eliminate NETs which are composed of dsDNA and histone proteins.^[^ ^34^ ^]^ In another experiment, neutrophils were stimulated with LPS for 4 h and then nanomaterials were added. The results showed that B‐P_M_, PG1, and DNase I effectively scavenged LPS‐induced NETs, with the highest efficacy in the B‐P_M_ group (Figure 4G,H). This outcome was further validated by SYTOX Green staining, which was used to label extracellular dsDNA and dead cell dsDNA (Figure S10, Supporting Information). Additionally, the generated NETs were collected and then incubated with nanomaterials, and the dsDNA content in NETs was quantified by pico‐green assay.^[^ ^35^ ^]^ The results displayed that the NETs binding efficacy of B‐P_M_ was higher than PG1 (Figure 4I), and B‐P_M_ also exhibited superior inhibition of NETs‐induced TLR9 activation compared to PG1 (Figure 4J).

In addition to the activation of inflammatory pathways, NETs were also reported to disrupt tight epithelial connections.^[^ ^36^ ^]^ In this study, we incubated NETs with airway epithelial cells to test whether the functionalized nanosheets could recover these detrimental effects. The expression of IL‐6, IL‐8, IL‐17A, and TNF‐α was upregulated in BEAS‐2B cells upon exposure to NETs. However, the addition of B‐P_M_ effectively inhibited this NETs‐induced inflammatory response (Figure S11A–D, Supporting Information). Furthermore, it was observed that NETs caused damage to primary airway epithelial cells and reduced the expression of E‐cadherin and Occludin, both crucial for maintaining the structural and functional integrity of the epithelium. However, B‐P_M_ demonstrated a robust capacity to reverse this detrimental effect and promote the expression of E‐cadherin and Occludin in NETs‐treated epithelial cells (Figure S11E,F, Supporting Information). These findings suggested that B‐P_M_ could not only inhibit NET formation but also scavenge the pre‐formed NETs. In this way, the positive feedback loop induced by cfDNA and NETs in airway inflammation was effectively alleviated. In addition, both suppression effects in the above experiments were more effective in the B‐P_M_ group than the PG1 group, indicating the advantage of 2D nanoplatforms for inflammation modulation.

### 2.4 RONS Elimination by B‐P_M_

**Figure 5:**
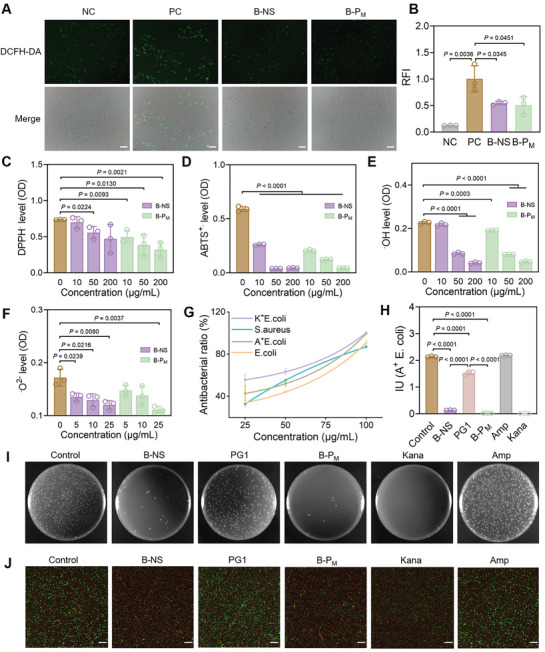
Antioxidant and antibacterial studies of B‐P_M_ in vitro. A) Representative DCFH‐DA staining images of the LPS‐treated BEAS‐2B cells after incubation with B‐NS and B‐P_M_. Scale bars: 50 µm. B) Quantitative analysis of relative fluorescence intensity (RFI) in (A). C–F) RONS reducing the ability of B‐NS and B‐P_M_ evaluated by C) DPPH·, D) ABTS^+^·, E) ·OH and F) ·O^2−^ level. G) Antibacterial effects of B‐P_M_ on E. coli, A^+^ E. coli, K^+^ E. coli, and S. aureus. H) The concentration of A^+^ E. coli liquid after 24h treatment of B‐NS, PG1, B‐P_M_, ampicillin (Amp), and kanamycin (Kana). IU: international unit. I) Photographs of bacterial colonies formed by A^+^ E. coli treated with B‐NS, PG1, B‐P_M_, Kana, and Amp for 24 h. J) Live/dead staining (green/red) of A^+^ E. coli treated with B‐NS, PG1, B‐P_M_, Kana, and Amp. Scale bars: 50 µm. Data are presented as mean ± SD (n = 3, Student's t‐test, two‐tailed).

In addition to cfDNA, RONS were also excessively generated in the injured tissues, contributing to the subsequent uncontrolled inflammatory response. Due to the antioxidant property of boron ester bonds in B─NS,^[^ ^27^, ^37^ ^]^ the RONS reduction properties of B‐NS and B‐P_M_ were also studied using DCFH‐DA probes. ROS was generated in BEAS‐2B cells by LPS stimulation, and the DCFH‐DA test showed that B‐P_M_ had a similar ROS scavenging ability to B‐NS (Figure S12, Supporting Information), indicating that PG1 modification did not reduce the ROS reduction capacity. Quantification of fluorescence intensity also demonstrated a significant decline in ROS level after the addition of B‐NS and B‐P_M_ (52% and 51%, respectively) (**Figure** **5**A,B). In addition, the RONS reduction effects of B‐NS and B‐P_M_ were quantitatively measured using DPPH· assay, ABTS^+^ assay, ·OH assay, and ·O^2−^ assay, respectively. Similar to the DCFH‐DA assay, B‐P_M_ also showed a similar capacity to reduce DPPH·, ABTS^+^, ·OH, and ·O^2−^ compared to B‐NS (Figure 5C–F). These findings suggested that B‐P_M_ could serve as an effective RONS scavenger for airway inflammation modulation.

### 2.5 Antibacterial Efficacy of B‐P_M_

Although the antibacterial properties of B‐NS have been reported due to the affinity between boron dihydroxyl groups and key components such as LPS or peptidoglycan (PGN) in bacteria,^[^ ^27^, ^37^ ^]^ it remains unclear whether the interaction was affected by PG1 coverage. In this study, the antibacterial properties of B‐NS, PG1, and B‐P_M_ were evaluated against four bacterial strains: *Escherichia coli* (*E. coli*), ampicillin‐resistant *Escherichia coli* (A^+^ *E. coli*), kanamycin‐resistant *Escherichia coli* (K^+^ *E. coli*) and *Staphylococcus aureus* (*S. aureus*). The results indicated that the antibacterial activity of B‐P_M_ increased along with the increase in concentration and almost approached 100% against all four bacterial strains at 100 µg mL^−1^ (Figure 5G). Fascinatingly, B‐P_M_ treatment exhibited a comparable anti‐bacterial effect to antibiotics for normal bacterial strains and was also effective against drug‐resistant bacteria (Figure 5H; Figure S13A–C, Supporting Information). Unexpectedly, B‐P_M_ displayed higher antibacterial capabilities compared to B‐NS, potentially attributed to the positive charge of B‐P_M_ facilitating electrostatic binding with bacteria. There was almost no colony formation on the Luria–Bertani (LB) plate in the B‐NS and B‐P_M_ treated A^+^ *E. coli*, while the number of colonies in the PG1 group was much higher, which is similar to the experimental results with *S. aureus* (Figure 5I; Figure S13D, Supporting Information). Furthermore, live/dead staining also revealed a significant percentage of dead bacteria in A^+^ E. coli treated with B‐NS and B‐P_M_ (Figure 5J). The antibacterial effect of B‐NS is due to the binding to bacterial cell wall components such as LPS/PGN, resulting in the breakdown of bacterial structure.^[^ ^27^ ^]^ The bactericidal mechanism of nanosheets was also confirmed by a scanning electron microscope (SEM), which records the morphological changes of bacteria. A^+^ *E. coli* displayed obvious wrinkles and shrinkage after 24 h of incubation with B‐NS or B‐P_M_, indicating the bacterial membrane rupture induced by nanosheets (Figure S13E, Supporting Information). These findings highlighted the superior antibacterial ability of B‐P_M_, which is crucial for the treatment of airway disorders.

### 2.6 Biosafety and Biodistribution of B‐P_M_

To further study the in vivo therapeutic effect of B‐P_M_, a biosafety evaluation with a high dosage (5 mg kg^−1^) of nanosheets was conducted. After 5 consecutive intranasal administrations (once per day), important organs (heart, liver, spleen, lung, kidney) and peripheral blood were collected from mice. Hematoxylin‐eosin (H&E) staining showed that B‐NS, PG1, and B‐P_M_ did not cause significant pathological damage to these organs (Figure S14A, Supporting Information). In addition, no significant differences in aspartate aminotransferase (AST), alanine aminotransferase (ALT), creatinine (CREA), and urea (URE) levels were recorded between the groups (Figure S14B–E, Supporting Information), indicating the favorable in vivo biocompatibility of these functionalized nanosheets.

**Figure 6:**
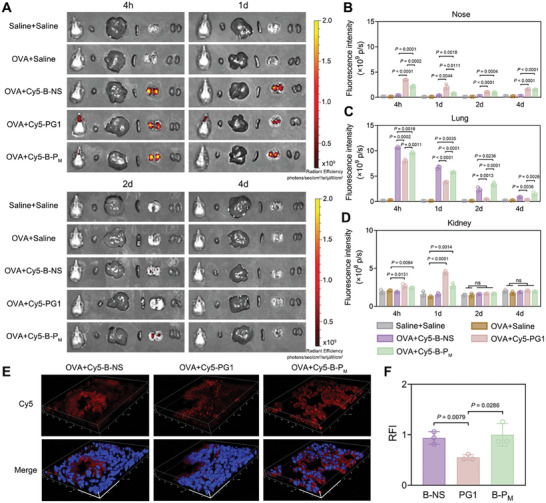
Biodistribution of B‐NS, PG1, and B‐P_M_ after intranasal treatment. A) Ex vivo fluorescence imaging of maxillary bones and major organs (heart, liver, spleen, lungs, and kidneys in sequence) at 4 h, 1 d, 2 d, and 4 d. B–D) Quantification of fluorescence intensity from noses, lungs, and kidneys in (A). E) Representative DAPI and Cy5 co‐staining 3‐D images of lungs at 1 d after treatments. Scale bars: 50 µm×50 µm×10 µm. F) Quantitative analysis of Cy5 fluorescence intensity in (E). Data are presented as mean ± SD (n = 3, Student's t‐test, two‐tailed).

Subsequently, we conducted biodistribution studies in ovalbumin (OVA)‐treated model mice to evaluate the accumulation of nanomaterials at the inflammatory sites. B‐NS, PG1, and B‐P_M_ were labeled with Cy5 and administered intranasally. Four hours later, significant enrichment of fluorescent signals from B‐NS and B‐P_M_ was observed in the lungs of mice, whereas PG1 preferentially accumulated in the nasal region (**Figure** **6**A–C). The accumulation of B‐P_M_ in the lungs was still observed after one day, ≈1.5 times higher than that of PG1, and gradually decreased over time, essentially disappearing at 4 d after treatment (Figure 6A–C). The fluorescence signals were also detected in the livers and kidneys at both 4 h and 1 day (Figure 6D; Figure S15A, Supporting Information), indicating that B‐P_M_ was metabolized and excreted from the livers and kidneys. In addition, no significant fluorescence differences were observed in the hearts, spleens, or blood (Figure S15B–D, Supporting Information). Confocal laser scanning microscopy (CLSM) imaging of lung sections from treated mice also revealed deeper penetration into lung tissues by B‐NS and B‐P_M_ compared to PG1 (Figure 6E,F; Figure S16, Supporting Information). These findings suggested that B‐P_M_ exhibited favorable biocompatibility and targeted accumulation in the airway tissues of model mice.

### 2.7 B‐P_M_ Ameliorated Airway Inflammation In Vivo

**Figure 7:**
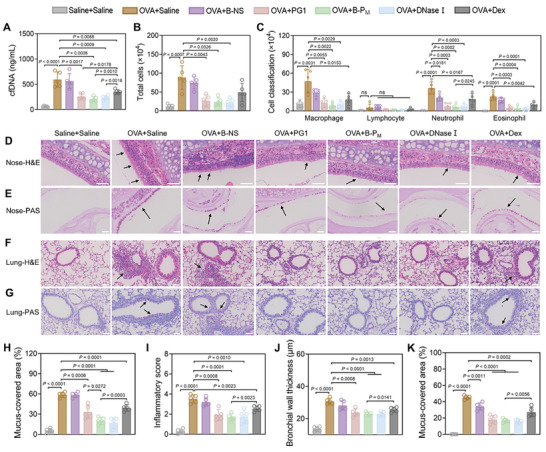
B‐P_M_ alleviated the airway inflammation in model mice. A) cfDNA concentration in BALF of mice in each group. B) Total cell number in BALF of mice in each group. C) The number of macrophages, lymphocytes, neutrophils, and eosinophils in BALF of mice in each group. D,E) Representative images of (D) H&E staining and (E) PAS staining of the mice nasal mucosa in each group. F,G) Representative images of (F) H&E staining and (G) PAS staining of the mice lung section in each group. (Arrows indicate epithelial damage in Nose‐H&E staining images and inflammatory cell infiltration in Lung‐H&E staining images, and point to mucus in PAS staining images. Scale bars: 50 µm.) H) Quantification of mucous formation in (E). I) The calculated inflammatory score is based on the infiltration of immune cells in (F). J) Quantification of the bronchial wall thickness in (F). K) Quantification of mucous formation in (G). Data are presented as mean ± SD (n = 5, Student's t‐test, two‐tailed).

Based on the therapeutic effect of B‐P_M_ observed in vitro, a mouse model with airway inflammation was established to evaluate its in vivo therapeutic efficacy. The mice were sensitized by intraperitoneal (i.p.) injection of OVA combined with complete Freund's adjuvant (CFA) on Day 0, Day 7 and challenged by intranasal (i.n.) instillation of OVA on Day 14–16. The experimental mice were treated with saline (i.n.), B‐NS (i.n.), PG1 (i.n.), B‐P_M_ (i.n.), DNase I (i.n.), and dexamethasone (Dex) (i.p.) 4 h after each challenge, respectively. The dosage of B‐NS, PG1, and B‐P_M_ (50 µg per mouse) was adopted based on our preliminary experiments, and the dosage of DNase I (20 IU per mouse) and Dex (1 mg kg^−1^ body weight) was referred to the previous publications.^[^ ^38^, ^39^, ^40^ ^]^ Compared to the saline‐treated sham group, the cfDNA level increased ≈10 times in bronchoalveolar lavage fluid (BALF) of the OVA‐treated inflammation group, along with an 8‐fold increase in total cells in BALF. After treatment, both the cfDNA level and total cells declined, with the most notable reduction observed in the B‐P_M_‐treated group (cfDNA: 593.9 ± 150.6 vs 205.8 ± 51.47 ng mL^−1^, total cells: 88.44 ± 31.37×10^4^ vs 24.18 ± 11.11×10^4^ cells) (**Figure** **7**A,B). Cell classification and counting based on bright‐field microscopy also showed a reduction in the number of macrophages, eosinophils, and neutrophils in BALF following B‐P_M_ treatment (Figure 7C). H&E staining also demonstrated PG1, B‐P_M_, DNase I, and Dex treatment attenuated inflammatory cell infiltration both in the nasal mucosa and lung tissues and restored tracheal wall thickness. Interestingly, B‐P_M_ exhibited similar effects to DNase I and was more effective than Dex (Figure 7D,F,I,J). Furthermore, periodic acid‐Schiff (PAS) staining indicated decreased mucus production in the nasal mucosa and lungs post‐treatment with PG1, B‐P_M_, DNase I, and Dex, which is beneficial to alleviate the overgeneration of snot and sputum of patients with rhinosinusitis or asthma. Notably, B‐P_M_ also exhibited similar mucus clearance efficacy as DNase I (Figure 7E,G,H,K). Due to the limitations of DNase I in clinical applications, the multifunctional B‐P_M_ is promising to be developed as a novel anti‐inflammation nanomedicine for airway disorders.

### 2.8 B‐P_M_ Attenuated NET Formation and Immune Cell Infiltration in the Airway

**Figure 8:**
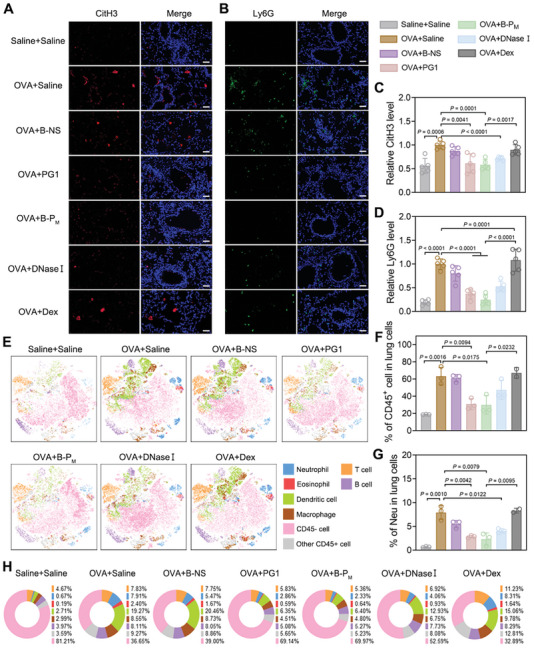
B‐P_M_ ameliorated the NET formation and immune cell recruitment in the lungs of model mice. A,B) Representative images of (A) CitH3 and (B) Ly6G immunostaining of the lungs. Scale bars: 50 µm. C,D) Quantitative analysis of (C) CitH3 and (D) Ly6G (n = 5). E) Visualization of t‐SNE analysis of various types of cells in the lung (Neu, Eos, T cells, B cells, DC, Mac, etc.). F,G) Percentage of (F) CD45+ cells and (G) neutrophils in total lung cells of each group (Dex: n = 2; other groups: n = 3). H) The pie chart of (E). Data are presented as mean ± SD (Student's t‐test, two‐tailed).

As excessive NETs played a pivotal role in the uncontrolled inflammatory response in airway disorders, we would examine if B‐P_M_ could successfully suppress NET formation in vivo. Immunofluorescent staining for CitH3 and lymphocyte antigen 6G (Ly6G) was conducted on mouse lung sections and nasal mucosa to measure neutrophil infiltration and NET formation levels, and significant elevations in CitH3 and Ly6G expression were observed in the inflammation group compared to the sham group. Following treatments with PG1, B‐P_M_, and DNase I, a decrease in neutrophil infiltration and NET formation both in nasal mucosa and lung tissues was noted, with the B‐P_M_‐treated group showing more pronounced alleviation (**Figure** **8**A–D; Figure S17A,D, Supporting Information). Similar to neutrophils and NETs, eosinophils could also degranulate and form eosinophil extracellular traps (EETs) under stimuli, especially in type II inflammation. The results of eosinophil cationic protein (ECP) immunofluorescent staining indicated that PG1, B‐P_M_, DNase I, and Dex treatment reduced EETs content compared to the inflammation group (Figure S17B,E, Supporting Information). However, IL‐5 immunofluorescence intensity did not show a significant reduction in the Dex group, confirming the restriction of glucocorticoid corticosteroids (GC) in the treatment of airway inflammatory disorders (Figure S17C,F, Supporting Information). Given that NETs and EETs could disrupt the epithelial integrity, the expression of epithelial markers (Cdh1, Ocln, and Tjp1) measured by quantitative real‐time polymerase chain reaction (qRT‐PCR) indicated rebounding levels of these indicators after treatment with PG1, B‐P_M_, DNase I, and Dex, suggesting protection to lung epithelium of inflammatory mice (Figure S18A–C, Supporting Information).

We also quantified the expression of various cytokines in the lungs of mice by qRT‐PCR, including type II cytokines IL‐4, IL‐5, and IL‐13 and non‐type II cytokines IL‐6 and IL‐17A. Treatment with PG1, B‐P_M_, and DNase I resulted in a reduction of both type II and non‐type II cytokine levels, while this alleviation was much weaker for experimental mice treated with Dex (Figure S19A–E, Supporting Information). Moreover, the expression level of Muc5ac was also decreased after treatment with PG1, B‐P_M_, and DNase I, which is consistent with the PAS staining results, indicating that mucus production was weakened in airway tissues (Figure S19F, Supporting Information). It is worth noting that B‐P_M_ demonstrated more robust therapeutic effects for inflammatory mice, indicating the advantage of a 2D planar backbone for the construction of cfDNA nano scavengers (Figure S19, Supporting Information). Consistent with qRT‐PCR results of gene expression, enzyme‐linked immunosorbent assay (ELISA) results also showed that B‐P_M_ treatment decreased the levels of cytokines (IL‐4, IL‐6, and IL‐17A) in BALF of mice with airway inflammation (Figure S20A–C, Supporting Information).

Due to its exceptional RONS scavenging effect in vitro, B‐P_M_ was evaluated for its antioxidant effect in vivo. The results showed that B‐NS and B‐P_M_ treatment significantly reduced malondialdehyde (MDA) content (from 2.15 ± 0.65 to 0.87 ± 0.17 nmol mL^−1^, and 0.78 ± 0.46 nmol mL^−1^), and restored superoxide dismutase (SOD) (from 2.40 ± 0.78 to 5.52 ± 0.51 U mL^−1^, and 5.01 ± 2.08 U mL^−1^) and reduced glutathione (GSH) (from 10.69 ± 3.0 to 66.85 ± 15.95 µg mL^−1^, and 52.83 ± 13.41 µg mL^−1^) levels, which was consistent with the previous antioxidant tests. However, the antioxidant efficacy is much less in the PG1 and DNase I treatment groups (Figure S21, Supporting Information).

Based on the above results, immune cell infiltration in the lungs of mice was comprehensively analyzed by multi‐channel flow cytometry. A significant increase of CD45^+^ cells in the lungs of inflammatory mice was observed compared to the saline‐treated group (more than 3 times) (Figure 8E,F). In more detail, the infiltration of neutrophils, eosinophils, macrophages, dendritic cells (DCs), T cells, and B cells within the total lung cells were all elevated, indicating the boosted airway inflammation in experimental mice (Figure 8G; Figure S22, Supporting Information). Fortunately, excessive infiltration of immune cells was largely ameliorated after B‐P_M_ treatment (neutrophils: decrease from 7.91% to 2.33%, eosinophils: decrease from 2.40% to 0.64%, macrophages: decrease from 8.55% to 4.80%, DCs: decrease from 19.27% to 6.40%, and T cells: decrease from 7.83% to 5.36%) (Figure 8G,H; Figure S22A–D, Supporting Information). In addition, B‐P_M_ treatment led to a reduction in the M2 subtype ratio of macrophages, which is also a significant indicator for inflammation modulation (Figure S23, Supporting Information). These results collectively confirmed the anti‐inflammatory properties of B‐P_M_, and confirmed the superiority of 2D nanostructure in the development of anti‐inflammation nanomedicine.

### 2.9 Transcriptome Analysis of the Lungs after Treatment

**Figure 9:**
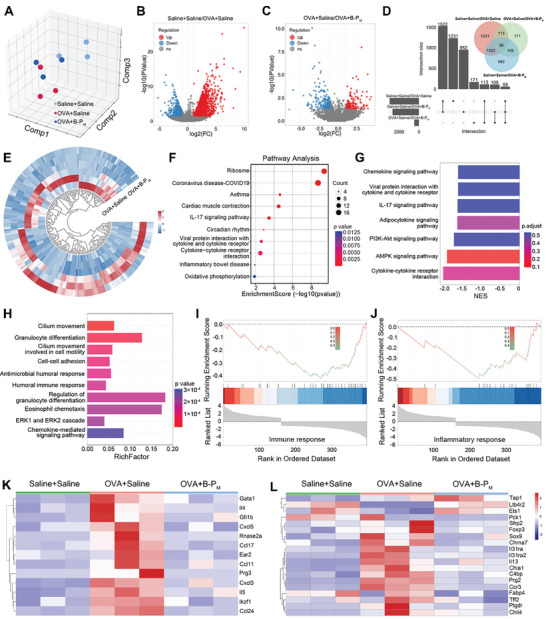
Transcriptome analysis of model mice lung tissues based on RNA sequencing. A) Principal component analysis (PCA) of the saline group, OVA group, and B‐P_M_‐treated group. B) Volcano maps based on the saline group and OVA group (Threshold: |log2(FC)| ≥ 1.0, *P* ≤ 0.05). C) Volcano maps based on OVA group and B‐P_M_‐treated group (Threshold: |log2(FC)| ≥ 1.0, *P* ≤ 0.05). D) UpSet plot and Veen map of DEGs between the saline group, OVA group, and B‐P_M_‐treated group. E) Heat map of the first 200 DEGs with significant changes between the OVA group and B‐P_M_‐treated group. F) KEGG pathway analyses of DEGs between the OVA group and B‐P_M_‐treated group. G) GSEA based on KEGG pathway analyses. H) GO term enrichment analyses of DEGs between the OVA group and B‐P_M_‐treated group. I,J) GSEA based on GO term enrichment analyses. K,L) Heat maps of DEGs associated with (K) granulocyte regulation and (L) immune response among saline group, OVA group, and B‐P_M_‐treated group (n = 3).

To gain a more comprehensive understanding of the therapeutic effect of B‐P_M_ on airway inflammation, we conducted transcriptome sequencing on mouse lungs. Principal component analysis (PCA) on the generated gene expression matrix (GEM) showed that RNA expression in the B‐P_M_ treatment group exhibited distinct transcriptomic differences from OVA‐treated mice while closer proximity to that in the sham group (**Figure** **9**A). By setting the threshold at |log2(FC)|≥1.0 and *P*≤0.05, a volcano map was generated to illustrate the variations in gene expression across different groups (Figure 9B,C). The Venn diagram illustrates the intersecting relationship of differentially expressed genes (DEGs) among the three groups (Figure 9D). The cluster heat map displayed the top 200 genes with the most significant differences between the B‐P_M_ treatment group and the inflammatory group (Figure 9E).

Then, Kyoto Encyclopedia of Genes and Genomes (KEGG) analysis was performed, and the pathway of DEGs enrichment after B‐P_M_ treatment is associated with asthma, IL‐17 signaling pathway, cytokine‐cytokine receptor interaction, oxidative phosphorylation, etc. (Figure 9F; Figure S24, Supporting Information). Further gene set enrichment analysis (GSEA) analysis of KEGG‐enriched pathways showed that the chemokine signaling pathway, IL‐17 signaling pathway, PI3K‐Akt signaling pathway, AMPK signaling pathway, and cytokine‐cytokine receptor interaction were down‐regulated after B‐P_M_ treatment (Figure 9G), prompting its therapeutic effect for airway inflammation. Gene Ontology (GO) analysis demonstrated that the DEGs were mainly involved in biological processes related to inflammatory response, including granulocyte differentiation, cell‐cell adhesion, anti‐microbial humoral response, humoral immune response, regulation of granulocyte differentiation, eosinophilic chemotaxis, ERK1/ERK2 cascade, chemokine‐mediated signaling pathways, etc. (Figure 9H; Figure S25, Supporting Information). Further GSEA analysis of GO biological processes indicated a down‐regulation of immune response and inflammatory response after B‐P_M_ treatment (Figure 9I,J). Based on the above results, we further analyzed the gene expression profiles associated with granulocyte regulation and immune response. The heat map illustrated a down‐regulation in the expression of these granulocyte‐positive regulatory genes and immune response genes in the B‐P_M_ treatment group compared to the inflammatory group (Figure 9K,L). These results confirmed that B‐P_M_ treatment effectively alleviated excessive airway inflammation, which is consistent with the previous experiments. In addition, GO analysis was performed on the DEGs between the B‐NS treatment group vs the inflammatory group and the PG1 treatment group vs the inflammatory group (Figures S26–S28, Supporting Information). The GO analysis of the DEGs in these two groups revealed a limited association with immune or inflammatory responses within the enriched biological processes (Figures S27, S28, Supporting Information). The results indicated the more robust anti‐inflammatory efficacy of B‐P_M_ compared to B‐NS and PG1, confirming the significance of multi‐targeting properties and unique 2D nanoscale geometry for nanomedicine development.

## 3 Discussion

Airway inflammatory disorders, including asthma, lung injury, CRSwNP, etc., typically manifest with symptoms such as nasal obstruction, reduced olfactory function, and airway hyperresponsiveness, with extremely adverse effects on quality of life.^[^ ^41^, ^42^ ^]^ Airway disorders have a substantial impact on patients’ quality of life and contribute to significant healthcare expenditures. Glucocorticoids (GC) are widely used for treating airway disorders in clinical both locally and systematically; however, the potential long‐term negative effects of repeated short courses of systemic GC must be weighed carefully against the potential benefits. Even short‐term use of oral corticosteroids (OCS) has been associated with an increased rate of sepsis, thromboembolism, and fractures. Moreover, systemic corticosteroid (SCS) therapy will increase the risk of pneumonia, cataracts, cardiovascular and cerebrovascular diseases, depression, and anxiety,^[^ ^43^ ^]^ highlighting the need for more effective therapies to manage these conditions.^[^ ^44^ ^]^

Several studies have reported elevated cfDNA levels in the airways of patients suffering from chronic airway inflammatory diseases (e.g., rhinitis, asthma, COPD).^[^ ^30^, ^45^, ^46^ ^]^ In our study, we first confirmed that cfDNA levels were negatively correlated with pulmonary functions and positively correlated to the pro‐inflammatory cytokines in ISS of asthma patients (Figure 1), suggesting a potential role for cfDNA in exacerbating lung inflammation and impairing pulmonary function in patients. In addition, the presence of NETs has been reported in various inflammation‐related diseases, often indicating the involvement of NETs in the associated pathological processes.^[^ ^47^ ^]^ Excessive and dysregulated NET formation can contribute to tissue damage and promote inflammation,^[^ ^48^ ^]^ and the use of DNase I has shown promise in alleviating inflammation.^[^ ^45^ ^]^ NAs‐binding polymers and nanomaterials can be applied as cfDNA scavengers in anti‐inflammatory treatments.^[^ ^49^, ^50^ ^]^ Our previous study found that cfDNA elimination could effectively reduce the levels of NETs and EETs, thereby offering a potential treatment for recalcitrant rhinosinusitis in model mice.^[^ ^30^, ^31^ ^]^ Unfortunately, the therapeutic effect was still unsatisfaction, which was attributed to the complicated inflammatory microenvironment, such as excessive RONS and bacterial infection.

Here, we developed novel multifunctional boron‐based nanosheets for airway inflammation treatment by targeting multiple inflammatory mediators, including excessive cfDNA, NETs, RONS, and bacterial infection. Such multi‐targeting properties of nanosheets provide more advantages compared to the current single‐targeting scavengers for anti‐inflammatory treatment (e.g., cfDNA scavengers or ROS scavengers).^[^ ^49^, ^51^, ^52^, ^53^ ^]^ Notably, functional nanosheets also achieved better therapeutic effects in model mice than dexamethasone, a commonly used glucocorticoid in clinical practice for airway inflammatory disorders. Although PG1 is a well‐established cationic cfDNA binding polymer, the quick excretion after administration due to the small molecular size makes it difficult to achieve the ideal therapeutic effect.^[^ ^54^ ^]^ B‐NS was selected in this study because nanosheets with 2D morphology and appropriate size are less likely to be phagocytosed by macrophages than spherical particles, and the appropriate size is also conducive to their penetration and accumulation in pathological airway tissues.^[^ ^28^, ^29^ ^]^ In addition, B‐NS exhibits outstanding antibacterial and antioxidant properties,^[^ ^27^ ^]^ which are particularly important for treating airway disorders. This is attributed to the fact that a large percentage of asthma patients are accompanied by uncontrolled bacterial infections, which brings new challenges to the clinical treatment.^[^ ^55^, ^56^ ^]^ Worse, the abuse of antibiotics often leads to the emergence of drug‐resistant bacteria, further increasing the difficulty of antibacterial therapy (Figure 2). Fascinatingly, PG1 coverage further improved the anti‐inflammation properties of functionalized nanosheets (Figures 4 and 5). B‐P_M_ nanosheets (200–300 nm) bound cfDNA with high affinity and suppressed cfDNA‐mediated TLR9 activation and NET formation. In addition, the B‐NS, PG1, B‐P_S_, B‐P_M_, and B‐P_L_ exhibited relatively low cytotoxicity, which is beneficial for the following biomedical applications. More importantly, PG1‐covered B‐NS exhibited a higher cfDNA‐scavenging capacity than PG1, highlighting the significance of the 2D nanoscale geometry for cfDNA scavenger development.

Since B‐P_M_ displayed more potent cfDNA binding ability compared to B‐P_S_ and B‐P_L_ and also exhibited sterling biocompatibility. In addition, the size of B‐P_M_ (200‐300 nm) is also suitable for penetration and accumulation in inflammatory airway tissues.^[^ ^28^ ^]^ Consequently, we studied the in vivo therapeutic effect of B‐P_M_ in a mouse model sensitized with OVA combined with CFA. This airway inflammation model combines both neutrophilic and eosinophilic characteristics to simulate severe respiratory inflammatory disorders, for which Dex therapy is not fully effective.^[^ ^57^ ^]^ Consistent with previous studies using DNase I,^[^ ^58^ ^]^ our work showed that B‐P_M_ could reduce cfDNA level and NETs production, and effectively relieve airway inflammation and mucus secretion. More importantly, B‐P_M_ exhibited a superior therapeutic effect compared to PG1 (Figures 7 and 8), which might be attributed to the higher accumulation in the inflammatory airway tissues. Moreover, the administration of B‐NS and B‐P_M_ decreased RONS levels in BALF, reducing overall inflammation. In contrast, the anti‐inflammation efficacy of the Dex‐treated group was much less than the mice treated with nanomaterials. Flow cytometry analysis of lung cells showed that the proportion of CD45^+^ cells was significantly reduced following B‐P_M_ treatment, and the percent of neutrophils, eosinophils, and macrophages all declined, consistent with cell count results of BALF.

Macrophages participate in pathways that promote type 1, 2, and 17 inflammation, which play critical roles in asthma phenotype/endotype and development,^[^ ^59^ ^]^ and effective clinical treatment of asthma may depend on the balance between M1 and M2 macrophages. It was confirmed that M1‐polarized macrophages were involved in the aggravation of allergic inflammatory responses during asthma. Although M2 macrophages secrete anti‐inflammatory cytokines and participate in the repair of damaged lungs, the persistent presence of these cells and the excessive generation of profibrotic factors could promote pathologies associated with allergic respiratory diseases, including airway remodeling and angiogenesis.^[^ ^59^ ^]^ In addition, adoptive transplanting of M2 macrophages into the lungs of asthmatic mice can effectively aggravate the severity of allergic airway inflammation,^[^ ^60^, ^61^ ^],^ and targeting M2 macrophages can be an alternative therapeutic strategy for asthma.^[^ ^62^ ^]^ These results jointly showed that the polarization of macrophages plays intricate regulatory roles in the development of asthma. In the results of flow cytometry, the proportion of macrophages with both M1 and M2 phenotypes significantly changed in the asthmatic mice compared to the sham group, indicating severe airway inflammation in model mice. However, the percent of macrophages, including both M1 and M2 phenotypes in the lungs of model mice after B‐P_M_ treatment, is closer to the control group, confirming the therapeutic effect of B‐P_M_ on airway inflammation. Previous studies have demonstrated that cfDNA/NETs can promote antigen presentation by DCs, which is closely related to allergic airway disorders.^[^ ^63^ ^]^ In line with other immune cells, our results indicated a reduction in the proportion of DCs after B‐P_M_ treatment, highlighting the regulatory effect of cfDNA removal on inflammatory immune response.

To obtain a more comprehensive analysis of the therapeutic effect of B‐P_M_ on airway inflammation, transcriptome analysis of the lungs of model mice was performed (Figure 9). It was found that B‐P_M_ modulated the immune response, granulocyte differentiation/chemotaxis, as well as chemokine and cytokine (and receptor) pathways, and some key inflammatory pathways were also significantly enriched and down‐regulated after treatment. For instance, the PI3K‐Akt signaling pathway, which is involved in the regulation of airway smooth muscle cell growth and proliferation, impacts airway hyperreactivity.^[^ ^64^, ^65^ ^]^ Furthermore, the PI3K signaling pathway is also implicated in neutrophil apoptosis and is critical for NET formation.^[^ ^66^, ^67^ ^]^ Regarding the IL‐17 signaling pathway, studies have shown that this IL‐17 family and its receptors play a central role in severe asthma,^[^ ^68^, ^69^ ^]^ contributing to structural alterations in epithelial cells and smooth muscle constriction, as well as being associated with resistance to steroids.^[^ ^70^ ^]^ Kim's study pointed out that the activation of the IL‐17 signaling pathway was associated with the release of histone proteins, which are the primary components of NETs,^[^ ^71^ ^]^ and its activation also leads to the recruitment of neutrophils and initiation of NET formation.^[^ ^72^, ^73^ ^]^ Other studies also showed the ERK1/ERK2 pathway could be activated by NETs,^[^ ^74^ ^]^ and the activation of ERK1/ERK2 can cause potent pro‐inflammatory effects, including inflammatory cell infiltration, goblet cell hyperplasia, and airway hyperreactivity in mouse lungs, which play an important role in severe airway diseases.^[^ ^75^ ^]^ In our experiment, all the above pro‐inflammatory pathways were suppressed after B‐P_M_ treatment, supporting the beneficial effects of B‐P_M_ on alleviating inflammation.

## 4 Conclusion

In summary, this study highlights the critical importance of targeting multiple inflammatory mediators in the treatment of severe airway inflammation and demonstrates the potential anti‐inflammation application of multifunctional boron‐based 2D nanoplatforms. The B‐P_M_ developed in this work exhibited multifaceted therapeutic capabilities, including cfDNA and NETs scavenging, antioxidant effects, and antibacterial properties. In addition, B‐P_M_ displayed a more robust cfDNA binding capacity than PG1, B‐P_S_, and B‐P_L_, indicating the significance of nanoscale geometry and size in the design of nanomedicine. More interestingly, B‐P_M_ effectively bound with NETs and prevented the NETs‐elicited immune cascade. The in vivo experiments displayed B‐P_M_ accumulated in the inflammatory airway tissues via nasal inhalation, effectively reducing cfDNA, NETs, and RONS levels. B‐P_M_ treatment mitigated immune cell infiltration, repaired damaged epithelium, decreased mucus production, and alleviated airway inflammation in model mice. Most importantly, the anti‐inflammation efficacy of B‐P_M_ outperformed the Dex, indicating the vast potential in future clinical applications. Overall, our results suggest that B‐P_M_ holds significant therapeutic potential for respiratory inflammation, and such a multiple‐targeting strategy also provides new insights into treating other inflammatory diseases.

## 5 Experimental Section

### Sputum Collection

A total of 94 subjects (24 healthy controls and 70 asthma patients) from the First Affiliated Hospital of Sun Yat‐sen University were included in this study. Patients with asthma were diagnosed according to the criteria of the Global Initiative for Asthma (GINA).^[^ ^2^ ^]^ Hypertonic saline was used to induce participants to cough up sputum, and 5 mL of peripheral blood was drawn. Then, the sputum plug was picked and weighed, and 0.1% dithiothreitol (DTT) was added four times to dissolve it. After the sputum was no longer viscous, it was filtered by cell sieve and centrifuged at 1000 rpm for 5 min to obtain the supernatant, which was ISS (induced sputum supernatant) and could be used for subsequent cfDNA determination and ELISA tests.^[^ ^76^ ^]^ Peripheral blood was used for subsequent neutrophil isolation. The protocol of clinical experiments was reviewed and approved by the Independent Ethics Committee (IEC) for Clinical Research and Animal Trials of the First Affiliated Hospital of Sun Yat‐sen University, approval number [2021]071. All the subjects had given their written informed consent.

### Animal Models

Animal experiments were approved by the ethics committee of the Sixth Affiliated Hospital of Sun‐Yat‐sen University (No. IACUC‐2022112801). C57BL/6JGpt female mice (6–8 weeks old) were purchased from GemPharmatech (Guangdong, China). The mice were housed in specific pathogen‐free (SPF) conditions with a 12‐h light/12‐h dark cycle at the Experimental Animal Center, The Sixth Affiliated Hospital, Sun Yat‐sen University. A mouse model with airway inflammation was established using modified protocols as previously described.^[^ ^57^ ^]^ The mice were randomly divided into seven groups (saline + saline, OVA + saline, OVA + B‐NS, OVA + PG1, OVA + B‐P_M_, OVA + DNase I, and OVA + Dex). These experimental mice were sensitized on day 0 and day 7 by intraperitoneally (i.p.) injecting 25 µg OVA in 100 µL of 0.9% saline mixed 1:1 with CFA. After sensitization, the mice were intranasally (i.n.) challenged with 50 µg OVA once per day from day 14 to day 16. 4 h after each challenge, 50 µg of B‐NS (i.n.), PG1 (i.n.) or B‐P_M_ (i.n.), 20 IU of DNase I (i.n.), or Dex (1 mg kg^−1^ body weight, i.p.) was administered for each experimental mouse. Mice sensitized and challenged with 0.9% saline served as a sham group. Mice were euthanized 24 h after the final treatment, and serum, bronchoalveolar lavage fluid (BALF), and lung tissues were collected for further experiments. There are five mice in each experimental group, except for the biodistribution study and mouse lung cell t‐SNE analysis experiments (three mice).

### Cell Counting and Classification in BALF

As previously outlined,^[^ ^77^ ^]^ BALF was obtained by infusing 0.6 mL of saline three times into the whole lungs via a tracheal cannula. Subsequently, BALF was centrifuged, the supernatant was stored at −80 °C, and the cell pellet was resuspended in 1 mL of PBS for cell counting using a Bio‐Rad automatic cell counter. Cell differential counting was performed using Wright–Giemsa staining, and cells were enumerated under a microscope.

### Histopathological and Immunofluorescence Staining

After collection of BALF, the right main bronchus was ligated, and the left lung was perfused and fixed with 4% paraformaldehyde for 24 h. Subsequently, the lung tissue was embedded in paraffin, sliced, and subjected to hematoxylin and eosin (H&E) or Periodic Acid‐Schiff (PAS) staining. The inflammatory degree of H&E staining was scored as follows:^[^ ^78^ ^]^ 0 for no inflammatory cell infiltration; 1 for a small amount of inflammatory cell infiltration; 2 for one layer of inflammatory cell infiltration; 3 for 2–4 layers of inflammatory cell infiltration; and 4 for more than 4 layers of inflammatory cell infiltration. Five view fields were selected randomly from each sample for statistical scoring. Immunofluorescence staining was performed using CitH3, Ly6G, IL‐5, and ECP antibodies. For the nasal mucosa analysis, the maxillary bones were first decalcified in EDTA solution for 3 weeks, followed by sectioning and staining using the same method. Five fields of view were selected randomly for fluorescence quantification with ImageJ software.

### Quantification of Redox Index In Vivo

The levels of GSH, MDA, and SOD in mouse BALF were measured following the protocols provided by the respective assay kits.

### Biosafety Evaluation In Vivo

The biosafety of nanomaterials was evaluated in healthy mice by intranasally administering B‐NS (100 µg), PG1 (100 µg), and B‐P_M_ (100 µg) for 5 consecutive days. One week after administration, the mice were euthanized, and major organs (heart, lungs, liver, spleen, kidneys) were subjected to H&E staining. Additionally, ALT, AST, CREA, and URE levels in serum were measured using an automatic biochemical analyzer.

### Biodistribution Assay In Vivo

Mice in the OVA group were intranasally administered with normal saline, Cy5‐labeled B‐NS (50 µg), PG1 (50 µg), and B‐P_M_ (50 µg), while a sham group treated with saline was also included. Imaging of the nose and major organs (hearts, lungs, livers, spleens, and kidneys) was conducted using the IVIS Spectrum system (PerkinElmer, USA) at 4 h, 1, 2, and 4 days post‐instillation. Simultaneously, serum samples were collected from mice to measure the concentration of nanomaterials in blood.

### Flow Cytometry Analysis

The lung tissue was gently ground and filtered through a 40 µm cell sieve, followed by the removal of red blood cells using an RBC lysis solution to obtain a single‐cell suspension. Subsequently, the cell types and status were analyzed via flow cytometry using surface (or intracellular) markers. The antibodies utilized in flow cytometry are detailed in Table S3 (Supporting Information).

### Transcriptome Sequencing and Analysis

TRIzol reagent was utilized for RNA extraction from lung tissues of the saline + saline group, OVA + saline group, OVA + B‐NS group, OVA + PG1 group, and OVA + B‐P_M_ group (n = 3). Subsequently, the sample library underwent quality assessment using Agilent 2100 Bioanalyzer, followed by paired‐end (PE) sequencing on these libraries utilizing Next‐Generation Sequencing (NGS) based on the Illumina sequencing platform. The differentially expressed genes between groups were screened by |log_2_(FC)|≥1.0 and *P*≤0.05. Bioinformatic analysis was performed using the Biodeep tools at https://www.biodeep.cn/home.

### Other Experiments

Other experimental details involved in this paper are provided in the Supporting Information.

### Statistical Analysis

Graph Pad Prism 8.0 and Origin 9.0 were used for data analysis and calculation of statistical significance. ImageJ was used for quantitative analysis of fluorescence intensity. The quantitative experiments were repeated with n = 3 or n = 5 per group, which was indicated in the figure legends. The results of in vitro experiments were obtained after independent replication at least three times. The data are presented as mean ± S.D. Statistical differences between experimental groups were assessed using unpaired two‐tailed Student's t‐test or Wilcoxon rank sum test. Spearman correlation analysis was used to analyze the correlation between the two variables.

## Conflict of Interest

The authors declare no conflict of interest.

## Supporting information



Supporting Information

## Data Availability

The data that support the findings of this study are available from the corresponding author upon reasonable request.
