# Supplementary material for: Multifunctional Boron‐based 2D Nanoplatforms Ameliorate Severe Respiratory Inflammation by Targeting Multiple Inflammatory Mediators
Source: Adv Sci (Weinh). 2025 Feb 14;12(13):2412626. doi: 10.1002/advs.202412626 (PMC11967860; doi:10.1002/advs.202412626)
Supplement: Supplementary file 1 — Supporting Information [file ADVS-12-2412626-s001.pdf]

ADVANCED SCIENCE
Open Access

Supporting Information

for *Adv. Sci.*, DOI 10.1002/advs.202412626

Multifunctional Boron-based 2D Nanoplatforms Ameliorate Severe Respiratory Inflammation by Targeting Multiple Inflammatory Mediators

*Changyi Xu, Ming Liu, Xinran Xie, Zhixin Li, Yuefei Zhu, Yang Ye, Mengya Du, Suhua Hu, Tianrun Liu, Yubiao Guo, Weiping Wen, Huanliang Liu and Zhaoxu Tu**

Supporting Information

Multifunctional boron-based 2D nanoplatforms ameliorate severe respiratory inflammation by targeting multiple inflammatory mediators

*Changyi Xu†, Ming Liu†, Xinran Xie†, Zhixin Li, Yuefei Zhu, Yang Ye, Mengya Du, Suhua Hu, Tianrun Liu, Yubiao Guo, Weiping Wen, Huanliang Liu, Zhaoxu Tu**

C. Xu, M. Liu, X. Xie, Z. Li, Y. Ye, M. Du, T. Liu, W. Wen, Z. Tu
Department of Otolaryngology, The Sixth Affiliated Hospital, Sun Yat-sen University, Guangzhou, Guangdong 510655, China
E-mail: tuzhx@mail.sysu.edu.cn

C. Xu, S. Hu, H. Liu
Department of Clinical Laboratory, The Sixth Affiliated Hospital, Sun Yat-sen University, Guangzhou, Guangdong 510655, China

Y. Guo
Department of Pulmonary and Critical Care Medicine, The First Affiliated Hospital, Sun Yat-Sen University, Guangzhou, Guangdong 510655, China

W. Wen
Department of Otolaryngology, The First Affiliated Hospital, Sun Yat-sen University, Guangzhou, Guangdong 510655, China

C. Xu, M. Liu, X. Xie, Z. Li, Y. Ye, M. Du, S. Hu, T. Liu, W. Wen, H. Liu, Z. Tu
Biomedical Innovation Center, The Sixth Affiliated Hospital, Sun Yat-sen University, Guangzhou, Guangdong 510655, China

Y. Zhu
Department of Biomedical Engineering, Columbia University, New York, New York 10027, United States

Experimental

Materials: Magnesium boride (MgB_2, catalogue no. 553913), gluconic acid (catalogue no. G1951), 2-morpholinoethanesulfonic acid (MES, catalogue no. M3671), 1-(3-dimethylaminopropyl)-3-ethylcarbodiimide (EDC, catalogue no. E7750), lipopolysaccharide (LPS, catalogue no. L2880), 2′,7′-dichlorofluorescin diacetate (DCFH-DA, catalogue no. D6883), dexamethasone (Dex, catalogue no. D4902), Freund's Adjuvant, Complete (CFA, catalogue no. F5881) and ovalbumin (OVA, catalogue no. A5503) were purchased from Sigma-Aldrich (St. louis, USA). Ethylenediamine was bought from KESHI (Chengdu, CHN). Methyl acrylate methanol (catalogue no. A13128) was purchased from Alfa Aesar (Massachusetts, USA). MACSxpress® Whole Blood Neutrophil Isolation Kit (human) (catalogue no. 130-104-434) was obtained from Miltenyi (Köln, GER). Quant-iT™ PicoGreen™ Kit (catalogue no. P7589), SYTOX® Green nucleic acid stain (catalogue no. S7020), TRIzol® Reagent (catalogue no. 15596026), LIVE/DEAD® BacLight™ Bacterial Viability Kit (catalogue no. L7012) and DNase I (catalogue no. EN0521) were bought from ThermoFisher (Massachusetts, USA). Phosphate buffered saline (PBS, catalogue no. C10010500BT), Penicillin/Streptomycin (catalogue no. 15140122), TRYPSIN 0.25% ethylene diamine tetraacetic acid (EDTA, catalogue no. 25200072), DMEM high glucose medium (catalogue no. C11995500BT), RPMI 1640 medium (catalogue no. C11875500BT) and fetal bovine serum (FBS, catalogue no. 10099141) were purchased from GIBCO (Grand Island, USA). PneumaCult™- Ex Plus basal medium (catalogue no.05040), PneumaCult™-Ex Plus supplement (catalogue no.05040) and hydrocortisone (catalogue no. 07925) were bought from Stemcell (Vancouver, CA). Bovine serum albumin (BSA)-FITC (catalogue no. SF063), red blood cell lysing buffer (catalogue no. R1010-500), reduced glutathione (GSH) content assay kit (catalogue no. BC1175), malondialdehyde (MDA) content assay kit (catalogue no. BC0025) and superoxide dismutase (SOD) activity assay kit (catalogue no. BC0175) were provided by Solarbio (Beijing, CHN). HEK-Blue™ hTLR9 (HEK-TLR9) cell (catalogue no. hkb-htlr9), HEK-TLR3 cell (catalogue

no. hkb-htlr3), HEK-TLR7 cell (catalogue no. hkb-htlr7), HEK-TLR8 cell (catalogue no. hkb-htlr8) and QUANTI-Blue^{TM} solution (catalogue no. rep-qbs) was purchased from InvivoGen (Toulouse, FR). BEAS-2B cell was obtained from ATCC (Virginia, USA). Cell counting kit-8 (CCK8, catalogue no. K1018) was obtained from APE×BIO (Houston, USA). 4',6-Diamidino-2'-phenylindole (DAPI, catalogue no. C1002) was bought from Beyotime (Shanghai, CHN). CitH3 antibody (catalogue no. ab281584) was purchased from Abcam (Cambridge, UK). All ELISA kits were provided by Neobioscience (Shenzhen, CHN). PrimeScript RT reagent (catalogue no. RR047A) and SYBR PreMix Ex Taq (catalogue no. RR820A) were purchased from Takara (Kusatsu, JPN). The primers used in this study (details were shown in Table S2) were provided by Tsingke (Beijing, CHN). IL-5 antibody (catalog no. PU142938) and ECP antibody (catalogue no. 55338) were bought from Abmart (Shanghai, CHN) and Proteintech (Wuhan, CHN), respectively. The antibodies involved in flow cytometry (details were shown in Table S3) were purchased from Biolegend (San Diego, USA).

Synthesis of PG1: 0.9 g (14.98 mmol) of ethylenediamine was dissolved in 5 mL of methanol and added dropwise to 10.3 g (119.6 mmol) of methyl acrylate methanol solution. The reaction mixture was stirred at room temperature for 24 hours and then distilled to remove methanol and excess methyl acrylate. The compound was dissolved in 10 mL of methanol and added dropwise to 37 g of ethylenediamine. After stirring for 24 hours, the solution was distilled to evaporate methanol and excess ethylenediamine to obtain PAMAM G0. Then 2 g of PAMAM G0 (dissolved in 10 mL of methanol) was added to 5.32 g (61.80 mmol) of methyl acrylate solution to react for 24 hours to synthesize PAMAM G0.5. Finally, the purified PAMAM G0.5 (2 g) was added dropwise to ethylenediamine (37 g) to obtain PAMAM G1 (PG1).

Synthesis of B-NS: 50 mg MgB_2 was added to 30 mL ddH_2O, and the reaction was performed at room temperature for 48 hours (inverted and mixed thoroughly during the reaction). After another 24 hours, the supernatant was collected and centrifuged at 1500 rpm for 45 min.[1] This

process was repeated twice, and the supernatant was lyophilized to obtain boron nanosheets (B-NS).

Synthesis of B-P: Gluconic acid (44 mg) was added to the B-NS (20 mg) solution, and the solution was stirred at room temperature for 24 hours (inverted and mixed thoroughly during the reaction). The 1,3-diol of gluconic acid could be conjugated to the boron dihydroxyl groups by esterification reaction. Excessive PG1 was added to modify the nanosheets via an amidation reaction with gluconic acid (with EDC in MES solution). The unreacted polymers were removed by dialysis (molecular weight cutoff of 10,000) for 48 hours to obtain PG1-covered B-NS (B-P). B-P was converted into B-P with small size (B-P_S), B-P with medium size (B-P_M), and B-P with large size (B-P_L) by ultrasound (500 W, 5 s on / 5 s off) for 30 min, 15 min, and 5 min, respectively.

Characterization of nanosheets: MALDI-TOF MS was detected by Bruker ultrafleXtrem. Zeta potential and dynamic light scattering (DLS) data were recorded on Brookhaven Zeta potential and particle size analyzer. Transmission Electron Microscope (TEM) was performed with Thermo Tecnai G2 Spirit T120 kV. Fourier transform infrared spectroscopy (FTIR) was measured on Vertex70-Hyperion3000. X-ray photoelectron spectroscopy (XPS) was recorded on ESCALAB Xi+.

cfDNA level determination: cfDNA concentration was measured using Quant-iT™ PicoGreen™ Kit according to the manufacturer’s protocol. Pico-green reagent was added to the experimental samples, and the cfDNA concentration can be calculated based on the fluorescence intensity (Ex = 480 nm; Em = 520 nm). The cfDNA scavenging ability of functionalized nanosheets was tested by the pico-green assay. Nanomaterials with a series of concentrations were added to the test solution with cfDNA (1 μg/mL), and then a pico-green reagent was added. The cfDNA binding efficacy was calculated by the fluorescence intensity variation.

Protein adsorption assay: Nanomaterials with a series of concentrations were mixed with BSA-FITC (100 μg/mL). The mixed solution was incubated at 37 °C for 30 min and then centrifuged at 11,000 rpm for 10 min. Subsequently, the supernatant was collected to detect its fluorescence intensity. Protein adsorption (PA) was calculated as follows: PA = (C1 - C2) / C1 × 100%. C1 represents the initial concentration of BSA, and C2 represents the concentration of BSA in the supernatant after centrifugation.

Hemolysis test: 1 mL of EDTA anticoagulated blood was centrifuged at 1000 rpm for 10 min, and the red blood cell pellet was washed with an appropriate amount of PBS, and the washing was repeated twice. After washing, 5-10 times PBS was added, followed by the addition of different concentrations of materials (dissolved in PBS), and ddH_2O was introduced to establish a positive control group. Following an incubation period at 37°C on a shaker for 4 hours, images were captured, and the absorbance was measured at 540 nm.

Isolation of primary airway epithelial cells: The airway tissue samples obtained by bronchoscopy were minced with sterile instruments. Subsequently, digestive enzymes (DNase I and collagenase 4) were added, and the digestion was carried out at 37°C shaker at 140 rpm for 20 min. The digested cell suspension was filtered through a 70 μm cell sieve and centrifuged at 300 g for 10 min at room temperature. Finally, the precipitates were resuspended in Ex-Plus Medium and continued to be cultured in a collagen I-coated T25 culture flask containing 5ml Ex-Plus medium at 37 °C in a humidified atmosphere with 5% CO_2.

Cell culture and treatment: BEAS-2B cells were cultured in a DMEM high glucose medium, with 1% penicillin-streptomycin and 10% fetal bovine serum. Primary airway epithelial cells (detailed extraction methods are provided in *Isolation of primary airway epithelial cells*) were cultured in PneumaCult™-Ex Plus basal medium, with 1% penicillin-streptomycin, 2% PneumaCult™-Ex Plus supplement, and 0.5% Hydrocortisone. Neutrophils were extracted using EDTA anticoagulated blood according to the MACSxpress® Whole Blood Neutrophil Isolation Kit (human) protocol, then cultured in RPMI 1640 medium, with 1% penicillin-

streptomycin and 10% fetal bovine serum. Cells were all cultured at 37 °C in a humidified atmosphere containing 5% CO_2. To obtain LPS-CM, BEAS-2B cells were first stimulated with different concentrations of LPS for 12 hours. Then the medium without LPS was replaced and cultured for an additional 12 hours to collect the resulting supernatant as LPS-CM. For cells incubated with NETs, neutrophils were first stimulated with LPS (5 μg/mL) to produce NETs, and then the medium was discarded and washed twice with DMEM. After that, DMEM with 10% FBS was added to rinse the bottom of the culture dish repeatedly until the bottom cells were basically blown away. The rinse fluid was collected and centrifuged at 1500 rpm for 5 min, and the supernatant was collected as the NET solution. Finally, NETs were introduced to cultured cells (BEAS-2B cells and primary airway epithelial cells) with or without B-P_M treatment; the cells incubated without NETs and B-P_M were established as control.

Cytotoxicity assay: BEAS-2B cells were seeded into 96-well plates, and nanomaterials with a series of concentrations (from 10 to 1000 μg/mL) were added to the cells after 24 hours. The cells were incubated in a 37°C, 5% CO_2 incubator for 48 hours. Finally, CCK8 was added, and the absorbance was detected at 450 nm to calculate cell viability.

TLR9 activation test: HEK-Blue^{TM} hTLR9 (HEK-TLR9) cells were cultured following the manufacturer's protocol. ISS, LPS-CM, or NETs (with or without nanomaterials) were added and co-incubated with HEK-TLR9 for 24 hours. The embryonic alkaline phosphatase secreted by HEK-TLR9 cells was detected with QUANTI-Blue^{TM} Solution to determine the degree of TLR9 activation. Similar methods were employed to detect TLR3, TLR7, and TLR8 activation.

Quantification of NET formation: Neutrophils were seeded into 24-well plates, with covered glass at the bottom of the wells. To assess the inhibitory effect of the nanomaterials on cfDNA-induced NET formation, neutrophils were stimulated with LPS-CM and co-cultured with 5 μg/mL of the PG1 or B-P_M for 24 hours. To evaluate the NETs elimination efficacy of the nanomaterials, 5 μg/mL of PG1 or B-P_M was added after LPS stimulation for 4 hours and co-cultured for an additional 24 hours. Immunofluorescence staining was performed using DAPI

and CitH3 and then observed by a confocal laser scanning microscope (CLSM). Additionally, SYTOX Green staining was applied and observed under a fluorescence microscope.

ELISA: The detection of protein concentration in experimental samples was carried out according to the ELISA kit protocol.

qRT-PCR: Total RNA was extracted by TRIzol and reverse transcribed into cDNA with the PrimeScript RT reagent kit. ABI QuantStudio 7 Flex and SYBR PreMix Ex Taq were used for qRT-PCR to quantify the mRNA level. The data were analyzed by the $2^{-\Delta\Delta Ct}$ method and reported as the relative variation (log2 transformed). The primers used in this step are listed in Table S2.

Intracellular RONS detection: BEAS-2B cells were seeded into 96-well plates (black) and allowed to attach to the bottom for 4-6 hours. Subsequently, the cells were stimulated with 10 μg/mL LPS for 24 hours, followed by co-culture with nanomaterials of varying concentrations for 3 hours. Finally, the DCFH-DA was added and incubated for 30 min. The OD value was measured by a plate reader (Thermo Scientific Varioskan Flash, Excitation = 495nm; Emission = 525nm), and fluorescence microscopy was used to obtain fluorescent images.

Determination of antioxidant capacity: To detect the DPPH· clearance capacity, 0.1 mM DPPH solution was prepared with anhydrous ethanol. Subsequently, 1mL of nanomaterials with different concentrations were cultured with 1 mL DPPH solution at room temperature under gentle shaking for 30 min, followed by centrifugation at 5000 rpm for 10 min. The absorption value of the supernatant was then measured at 517 nm. The DPPH mixed with an equal volume of ddH_2O was set as the control group.

To detect the $ABTS^{+}\cdot$ scavenging capacity, 7.4 mM ABTS, and 2.6 mM $K_2S_2O_8$ were mixed in equal proportion and reacted at room temperature for 12 hours in the dark. The mixture was then diluted with anhydrous ethanol to achieve an absorbance of approximately 0.7 at 734 nm, and the solution was the ABTS working liquid. Subsequently, 0.8 mL of ABTS working liquid was mixed with 0.2 mL of nanomaterials with different concentrations under gentle shaking for

6 min at room temperature. The absorption value was measured at 734 nm. The ABTS working liquid mixed with an equal volume of ddH_2O was set as the control group.

To detect the ·OH clearance capacity, 9 mM salicylic acid solution, 9 mM $FeSO_4$ solution, and 8.8 mM H_2O_2 solution were first prepared. Then, 30 μL of the three above-mentioned solutions and nanomaterials with different concentrations were added to the tubes, and the volume was filled to 450 μL with ddH_2O. During the same time, a blank group without H_2O_2 and a control group without nanomaterials were established, and both were supplemented with ddH_2O to reach a volume of 450 μL. The reactions were incubated in a water bath at 37°C for 15 min before measuring the absorbance at 510 nm.

To detect the $\cdot O^{2-}$ scavenging capacity, 1.2 mM xanthine solution, 0.05 unit/mL xanthine oxidase solution, and 1 mg/mL dihydroethidium (DHE) solution were first prepared. Subsequently, 40 μL xanthine oxidase solution and 40 μL xanthine solution were mixed in PBS at 37°C for 40 min to generate superoxide anions. Following this, 20 μL nanomaterials with different concentrations were added to the above solutions and incubated for 40 min. Finally, 100 μL DHE solution was added, and the OD value was measured (Excitation = 470 nm; Emission = 610 nm).

Antibacterial studies: E. coli, A^+ E. coli, and K^+ E. coli was cultured in Luria-Bertani (LB) medium or LB Agar. S. aureus was cultured in Nutrient Broth (NB) medium or Nutrient Agar (NA). Single colonies of each strain were selected for amplification, then diluted and co-cultured with nanomaterials with different concentrations for 24 hours. The colony formation of the bacterial suspension was quantified by measuring the absorbance at 600 nm using Nanodrop. Additionally, the bacterial suspension and nanomaterials with different concentrations were spread on agar plates and photographed after 24 hours.

For bacterial live/dead staining, the bacteria were incubated with 100 μg/mL of nanomaterials for 24 hours and then tested using LIVE/DEAD® BacLight™ Bacterial Viability Kits. In brief, the bacteria were concentrated by centrifugation and then incubated with

fluorescent dye (SYTO 9 and propidium) for 15 min. Then the bacteria were observed by CLSM (live: Ex = 480 nm, Em = 500 nm; dead: Ex = 490 nm, Em = 635 nm) after pipetting 5 μL of stained bacterial suspension onto a glass slide.

For SEM characterizations of bacteria, the bacteria were treated with 100 μg/mL of B-NS or B-P_M for 24 hours and then washed with PBS. Then, 2.5% glutaraldehyde solution was added for fixation, followed by washing with PBS and dehydration with ethanol, and the morphological changes of bacteria were analyzed by HITACHI SU8100.

Table S1. Characteristics of subjects

	Healthy subjects	Asthma patients	*p* value
Number	24	70	
Sex, M:F (%F)	8/16(66.7)	39/31(44.3)	0.0966
Age, yr	39.54±17.76	47.04±14.79	0.0838
BMI, kg/m^2	20.98±2.69	23.25±2.98	0.0007
Pulmonary function			
FEV1, % predicted	102.5±8.54	69.51±24.79	<0.0001
FVC, % predicted	101.8±10.61	89.75±19.51	0.0011
FEV1/FVC, %	87.14±6.44	68.03±14.04	<0.0001
Histamine PC20, mg/mL	na	1.64±2.11	-
FeNO, ppb	13.09±5.67	64.88±58.78	<0.0001
Blood			
Eosinophil rate, n (%)	1.72±0.91	5.06±4.23	0.0001
Neutrophil rate, n (%)	59.85±10.48	58.81±10.37	0.7461
Eosinophil count, n (10^9/L)	0.10±0.05	0.35±0.30	<0.0001
Neutrophil count, n (10^9/L)	3.95±1.53	4.08±1.57	0.6062
Leukocyte count, n (10^9/L)	6.39±1.39	6.76±1.89	0.4652
IgE, IU/mL	94.85±114.30	451.50±606.40	0.0012

Values are presented as mean ± SD. BMI: body mass index; FEV1, % predicted: forced expiratory volume at 1 s to predicted value ratio, FVC, % predicted: forced vital capacity to predicted value ratio; PC20, the dose at which FEV1 falls >20% of baseline FEV1; FeNO, fraction of exhaled nitric oxide.

Table S2. Primers used in qRT-PCR

Gene	Species	Type	Sequence
GAPDH	Human	Forward	GTCTCCTCTGACTTCAACAGCG
		Reverse	ACCACCCTGTTGCTGTAGCCAA
IL-6	Human	Forward	AGACAGCCACTCACCTCTTCAG
		Reverse	TTCTGCCAGTGCCTCTTTGCTG
IL-8	Human	Forward	ACTGAGAGTGATTGAGAGTGGAC
		Reverse	AACCCTCTGCACCCAGTTTTC
IL-17A	Human	Forward	CGGACTGTGATGGTCAACCTGA
		Reverse	GCACTTTGCCTCCCAGATCACA
TNF-α	Human	Forward	GAGGCCAAGCCCTGGTATG
		Reverse	CGGGCCGATTGATCTCAGC
E-cadherin	Human	Forward	GAAGGTGACAGAGCCTCTGGAT
		Reverse	GATCGGTTACCGTGATCAAAATC
Occludin	Human	Forward	ATGGCAAAGTGAATGACAAGCGG
		Reverse	CTGTAACGAGGCTGCCTGAAGT
Gapdh	Mouse	Forward	AGGTCGGTGTGAACGGATTTG
		Reverse	TGTAGACCATGTAGTTGAGGTCA
IL-4	Mouse	Forward	GGTCTCAACCCCCAGCTAGT
		Reverse	GCCGATGATCTCTCTCAAGTGAT
IL-5	Mouse	Forward	TCAGGGGCTAGACATACTGAAG
		Reverse	CCAAGGAACTCTTGCAGGTAAT
IL-13	Mouse	Forward	CCTGGCTCTTGCTTGCCTT
		Reverse	GGTCTTGTGTGATGTTGCTCA
IL-6	Mouse	Forward	AGACAGCCACTCACCTCTTCAG

		Reverse	TTCTGCCAGTGCCTCTTTGCTG
IL-17A	Mouse	Forward	TATCCCTCTGTGATCTGGGAAG
		Reverse	ATCTTCTCGACCCTGAAAGTGA
Muc5ac	Mouse	Forward	CAGGACTCTCTGAAATCGTACCA
		Reverse	AAGGCTCGTACCACAGGGA
Cdh1 (E-cadherin)	Mouse	Forward	GGTCATCAGTGTGCTCACCTCT
		Reverse	GCTGTTGTGCTCAAGCCTTCAC
Ocln (Occludin)	Mouse	Forward	TGGCAAGCGATCATACCCAGAG
		Reverse	CTGCCTGAAGTCATCCACACTC
Tjp1	Mouse	Forward	GTTGGTACGGTGCCCTGAAAGA
		Reverse	GCTGACAGGTAGGACAGACGAT

Table S3. Fluorescent antibodies used in flow cytometry

Antibody	Catalogue No.
Zombie Aqua™ Fixable Viability Kit	423101
FITC anti-mouse CD45	103108
PerCP/Cyanine5.5 anti-mouse CD3	100218
Brilliant Violet 605™ anti-mouse/human CD11b	101257
Alexa Fluor® 700 anti-mouse Ly-6G	127622
Brilliant Violet 421™ anti-mouse I-A/I-E	107632
PE/Cyanine7 anti-mouse CD11c	117317
APC anti-mouse F4/80	123115
APC/Cyanine7 anti-mouse CD86	105029
PE anti-mouse CD206 (MMR)	141706
Brilliant Violet 650™ anti-mouse CD19	115541
PE anti-mouse CD170 (Siglec-F)	155505

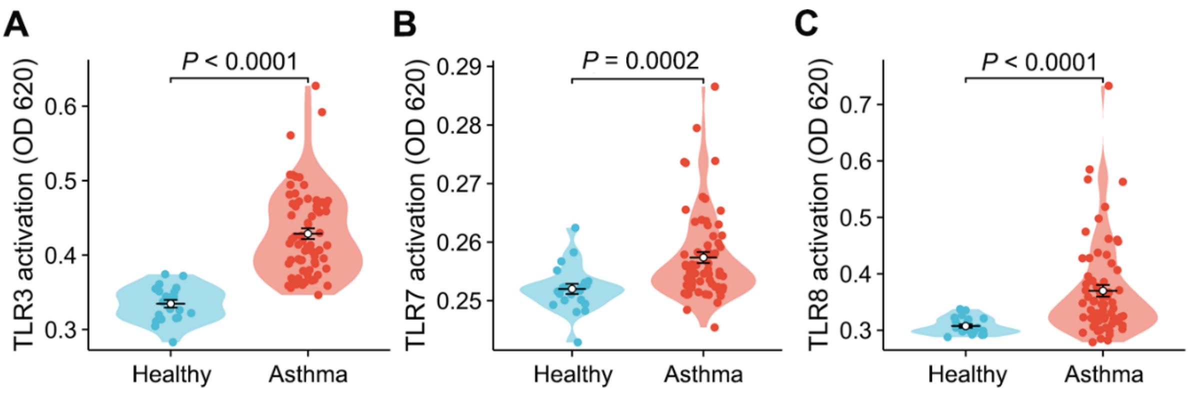

Figure S1. Activation of TLRs by ISS. Activation of (**A**) TLR3, (**B**) TLR7, and (**C**) TLR8 by ISS of healthy volunteers and asthma patients. Data are expressed as mean ± SEM (Control: n = 21; Asthma: n = 67. Wilcoxon rank sum test, two-tailed).

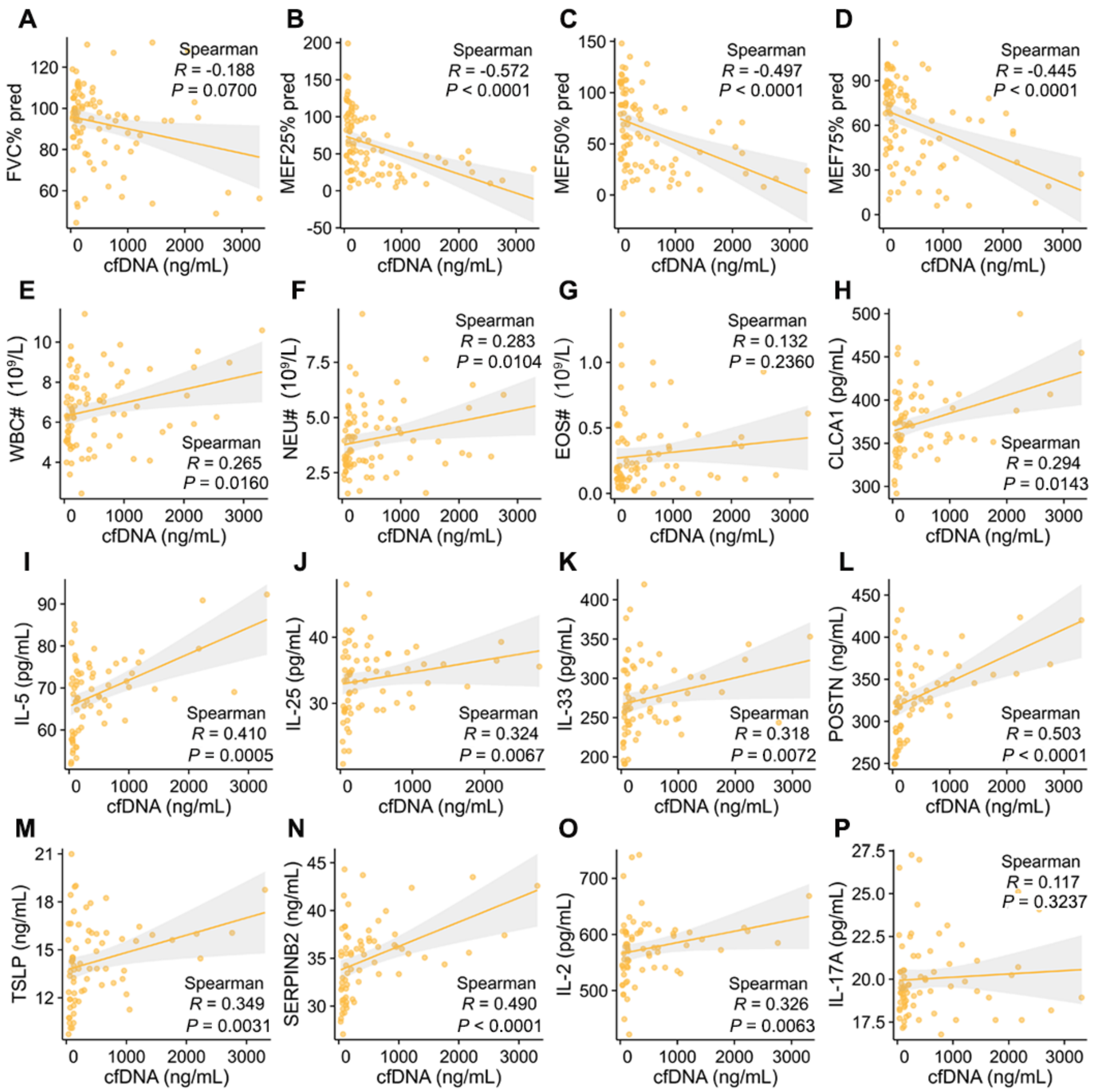

Figure S2. Correlation analysis of cfDNA with pulmonary function and inflammation level. Correlation of cfDNA in ISS with (**A**) FVC% pred (n = 94), (**B**) MEF25% pred (n = 92), (**C**) MEF50% pred (n = 92), (**D**) MEF75% pred (n = 92), and the contents of (**E**) white blood cells (n = 82), (**F**) neutrophils (n = 81) and (**G**) eosinophils (n = 82) in peripheral blood, and (**H**) CLCA1 (n = 69), (**I**) IL-5 (n = 69), (**J**) IL-25 (n = 70), (**K**) IL-33 (n = 70), (**L**) POSTN (n = 69), (**M**) TSLP (n = 70), (**N**) SERPINB2 (n = 69), (**O**) IL-2 (n = 69), (**P**) IL-17A (n = 73) protein concentration in ISS (Spearman correlation analysis).

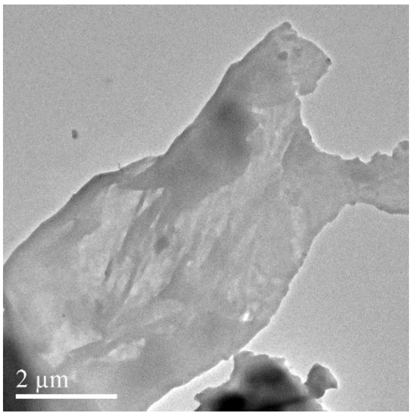

Figure S3. TEM of B-NS.

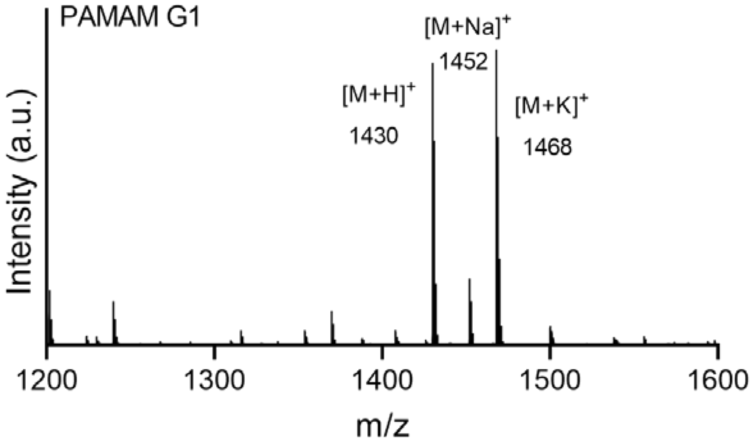

Figure S4. Mass Spectrometry of PAMAM G1.

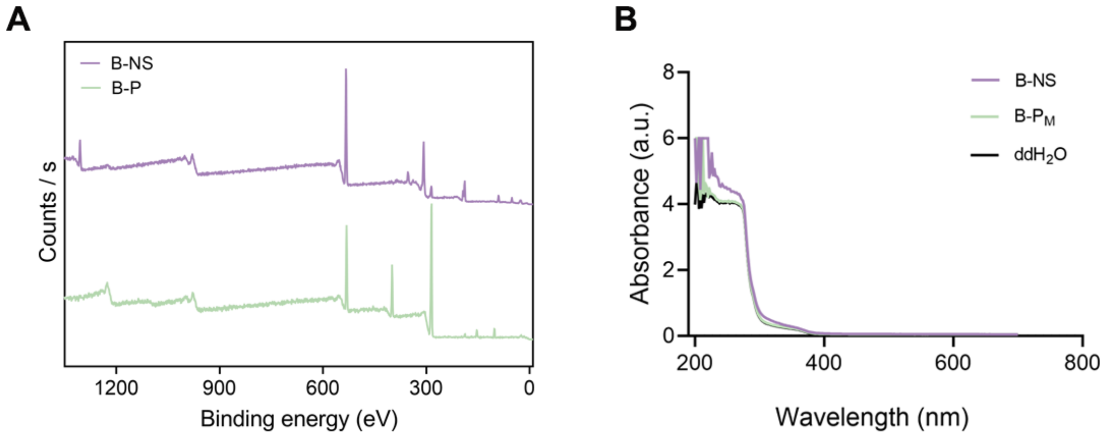

Figure S5. (**A**) XPS survey spectra of B-NS and B-P. (**B**) UV absorption spectra of B-NS and B-P$_M$.

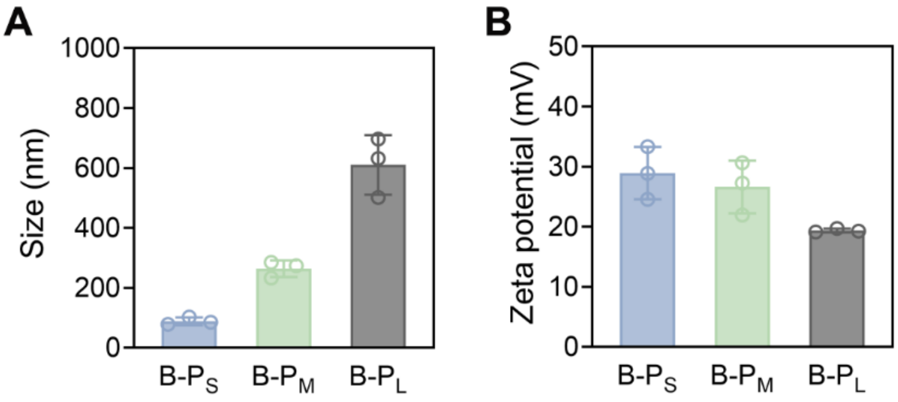

Figure S6. Size and zeta potential of B-P$_S$, B-P$_M$, and B-P$_L$. (**A**) The particle size of B-P$_S$, B-P$_M$, and B-P$_L$ measured by DLS. (**B**) The zeta potential of B-P$_S$, B-P$_M$, and B-P$_L$. Data are presented as mean ± SD (n = 3).

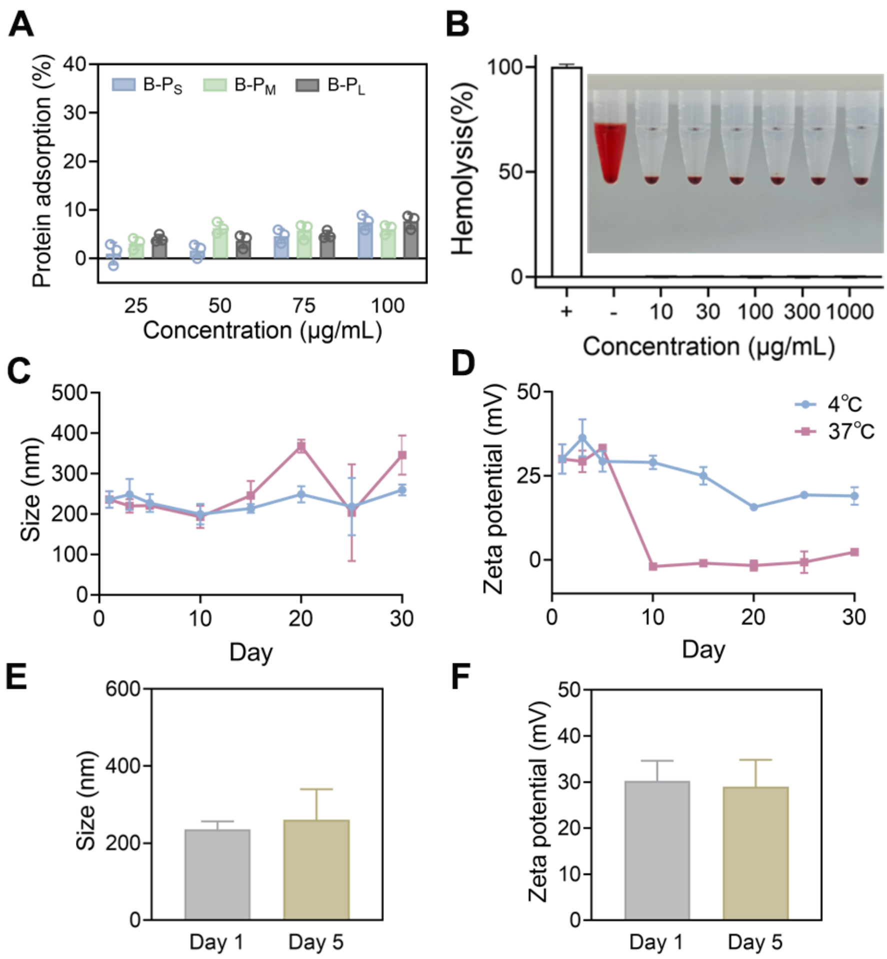

Figure S7. Biocompatibility and stability of B-P$_M$ *in vitro*. (**A**) Protein adsorption capacity of B-P$_S$, B-P$_M$, and B-P$_L$ after incubation with BSA. (**B**) The hemolytic ability of B-P$_M$ after incubation with red blood cells. (**C**, **D**) The change of (**C**) particle size and (**D**) zeta potential of B-P$_M$ at different temperatures within 30 days. (**E**, **F**) The (**E**) particle size and (**F**) zeta potential of B-P$_M$ in PBS after incubation for 1 day or 5 days. Data are presented as mean ± SD (n = 3).

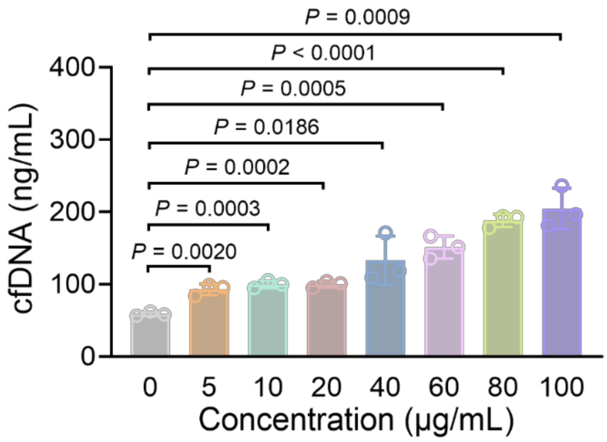

Figure S8. cfDNA release by BEAS-2B cells after LPS stimulation. cfDNA content in BEAS-2B culture medium after incubation with LPS at different concentrations. Data are presented as mean ± SD (n = 3, Student's t-test, two-tailed).

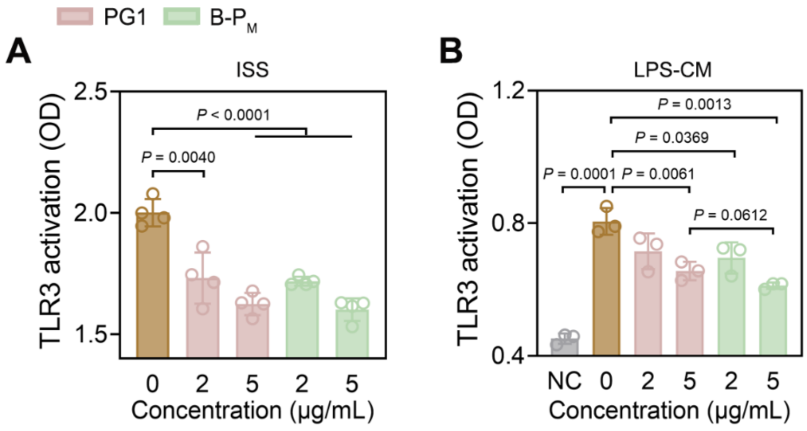

Figure S9. Suppression of TLR3 activation by B-P$_M$. (**A**, **B**) TLR3 activation of HEK-TLR3 cells induced by (**A**) ISS (n = 4) and (**B**) LPS-CM (n = 3) after incubation with PG1 and B-P$_M$. NC: negative control. Data are presented as mean ± SD (Student's t-test, two-tailed).

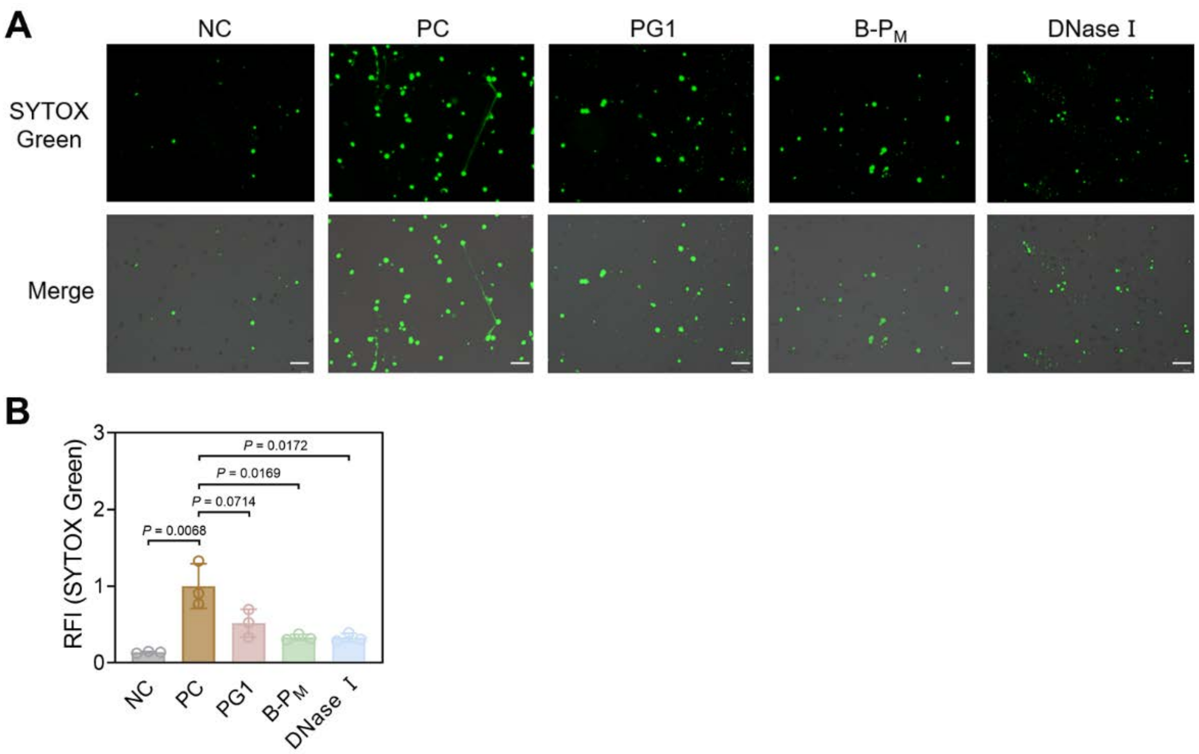

Figure S10. The suppression of NET generation by B-P$_M$. (**A**) Representative SYTOX Green fluorescent staining images of the NETs produced by LPS-treated neutrophils after incubation with PG1, B-P$_M$, and DNase I. Scale bars: 50 µm. (**B**) Quantitative analysis of fluorescence intensity in (**A**). NC: negative control. PC: positive control. Data are presented as mean ± SD (n = 3, Student's t-test, two-tailed).

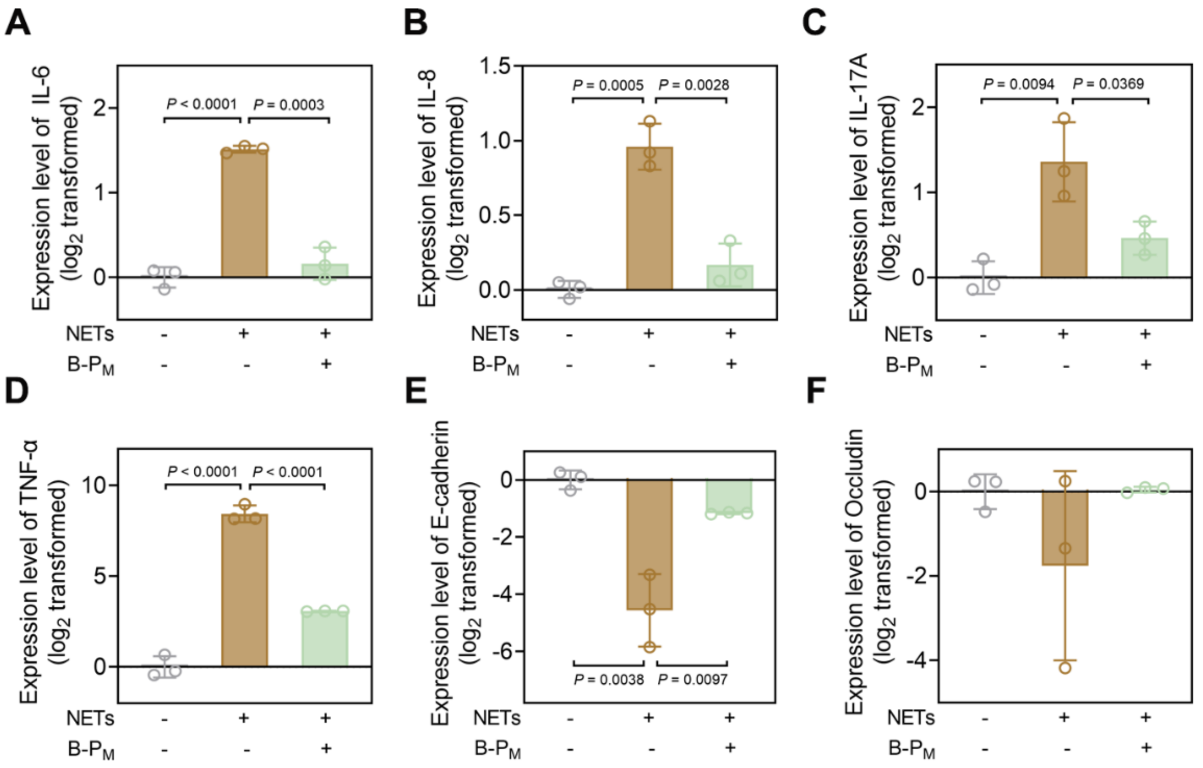

Figure S11. The alleviation of NETs-induced inflammation and epithelial cell damage by B-P$_M$ *in vitro*. (**A–D**) The expression levels (qRT-PCR) of (**A**) IL-6, (**B**) IL-8, (**C**) IL-17A and (**D**) TNF-α of BEAS-2B cells in each treatment group. (**E–F**) The expression levels (qRT-PCR) of (**E**) E-cadherin and (**F**) Occludin of primary airway epithelial cells in each treatment group. Data are presented as mean ± SD (n = 3, Student's t-test, two-tailed).

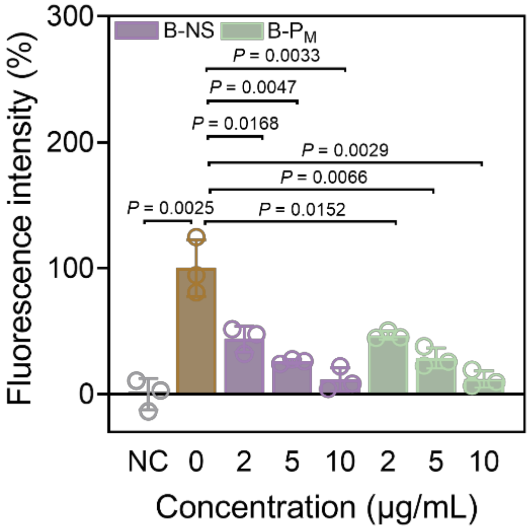

Figure S12. The effect of B-NS and B-P$_M$ on ROS clearance *in vitro*. Relative DCFH-DA fluorescence intensity of LPS-stimulated BEAS-2B cells treated with B-NS and B-P$_M$. Data are presented as mean ± SD (n = 3, Student's t-test, two-tailed).

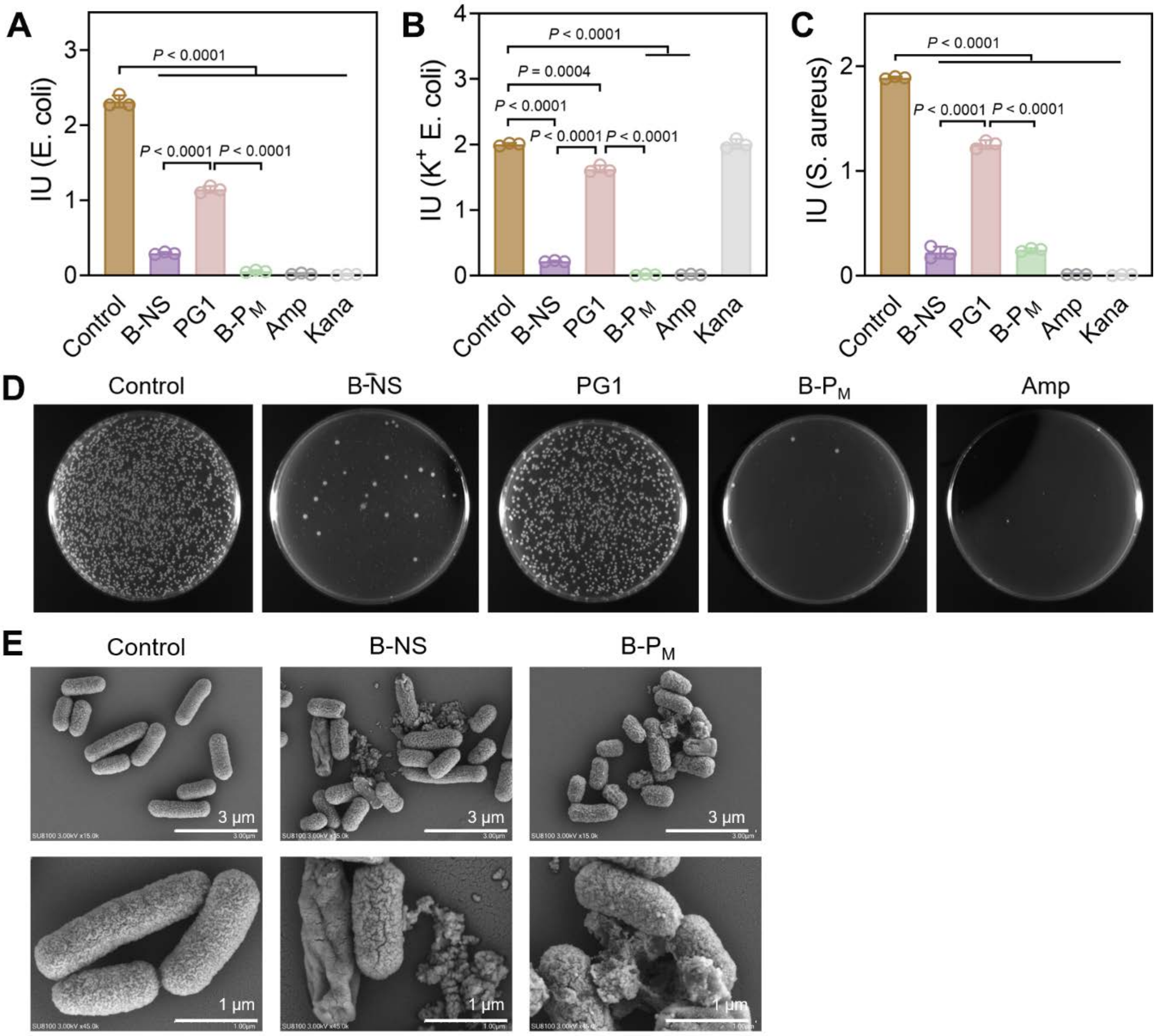

Figure S13. Antibacterial activity of B-NS, PG1, and B-P$_M$ *in vitro*. (**A-C**) The concentration of (**A**) E. coli, (**B**) K$^+$ E. coli, and (**C**) S. aureus liquid after 24 h treatment of B-NS, PG1, B-P$_M$, Amp, and Kana. (**D**) Photographs of bacterial colonies formed by S. aureus treated with B-NS, PG1, B-P$_M$, and Amp. (**E**) SEM images of A$^+$ E. coli subjected to B-NS and B-P$_M$. Scale bar (up)= 3 µm, scale bar (down) = 1 µm. Data are presented as mean ± SD (n = 3, Student's t-test, two-tailed).

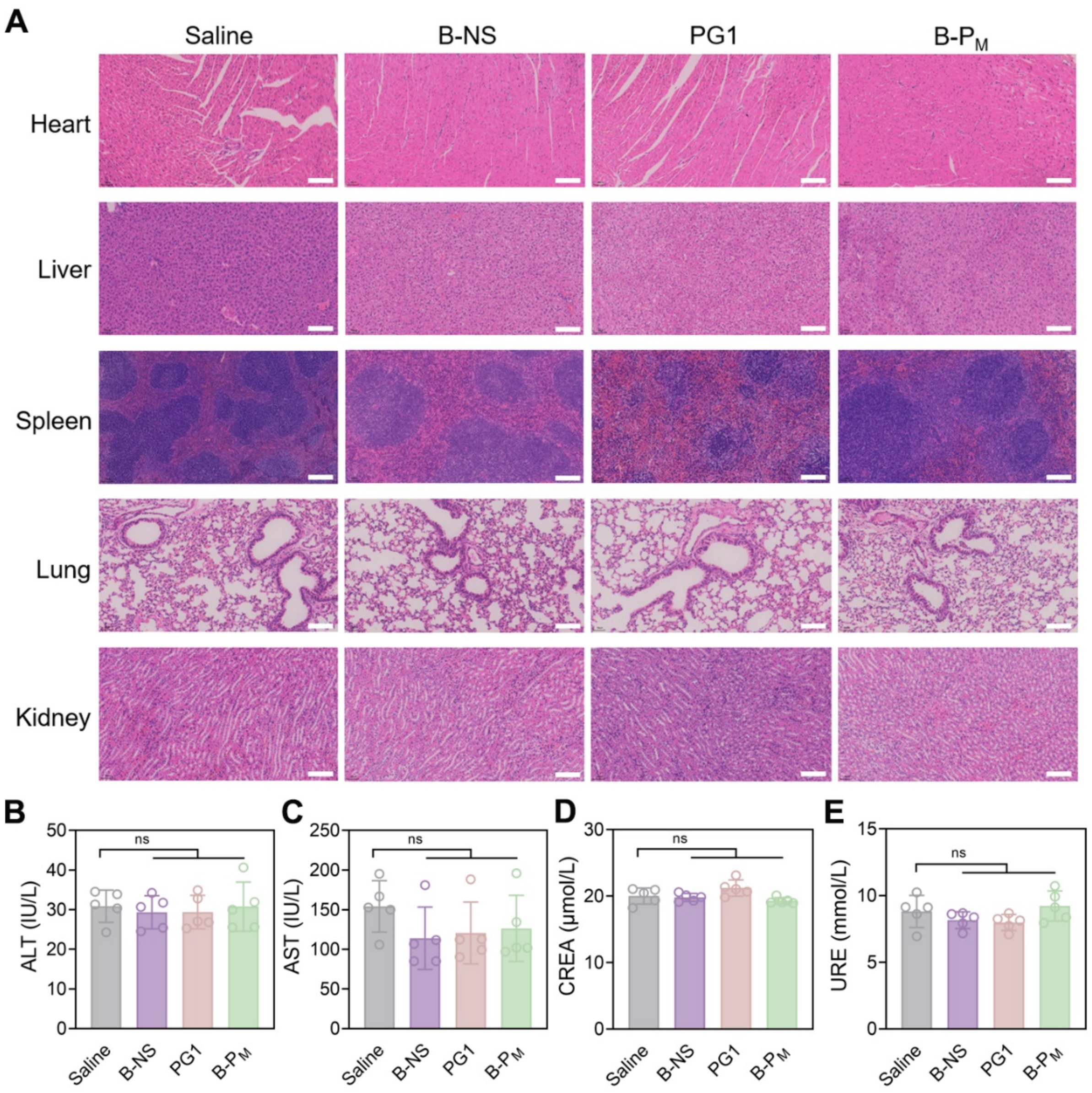

Figure S14. Biocompatibility of B-NS, PG1, and B-P$_M$ *in vivo*. (**A**) Representative H&E staining images of hearts, livers, spleens, lungs, and kidneys of mice treated with B-NS, PG1, and B-P$_M$. Scale bars: 100 μm. (**B**) ALT, (**C**) AST, (**D**) CREA, and (**E**) URE levels in the peripheral blood of mice in each group. Data are presented as mean ± SD (n = 5, Student's t-test, two-tailed).

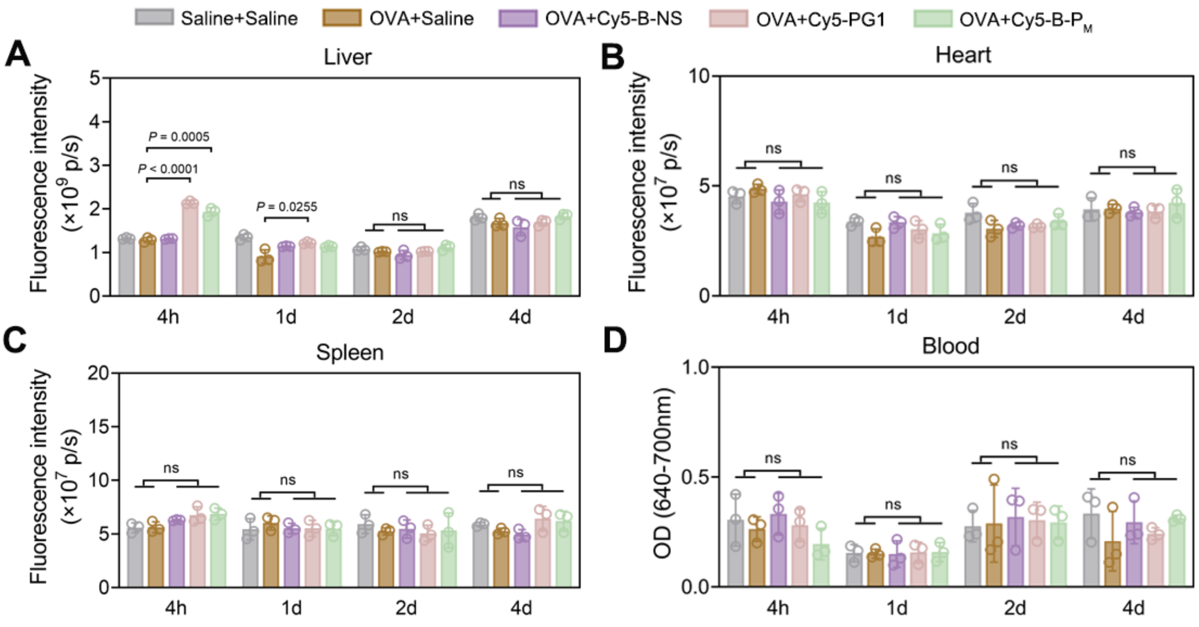

Figure S15. Biodistribution of B-NS, PG1, and B-P$_M$ after treatment. (**A–C**) Quantification of fluorescence intensity from (**A**) liver, (**B**) heart, and (**C**) spleen. (**D**) Fluorescence intensity in peripheral blood. Data are presented as mean ± SD (n = 3, Student's t-test, two-tailed).

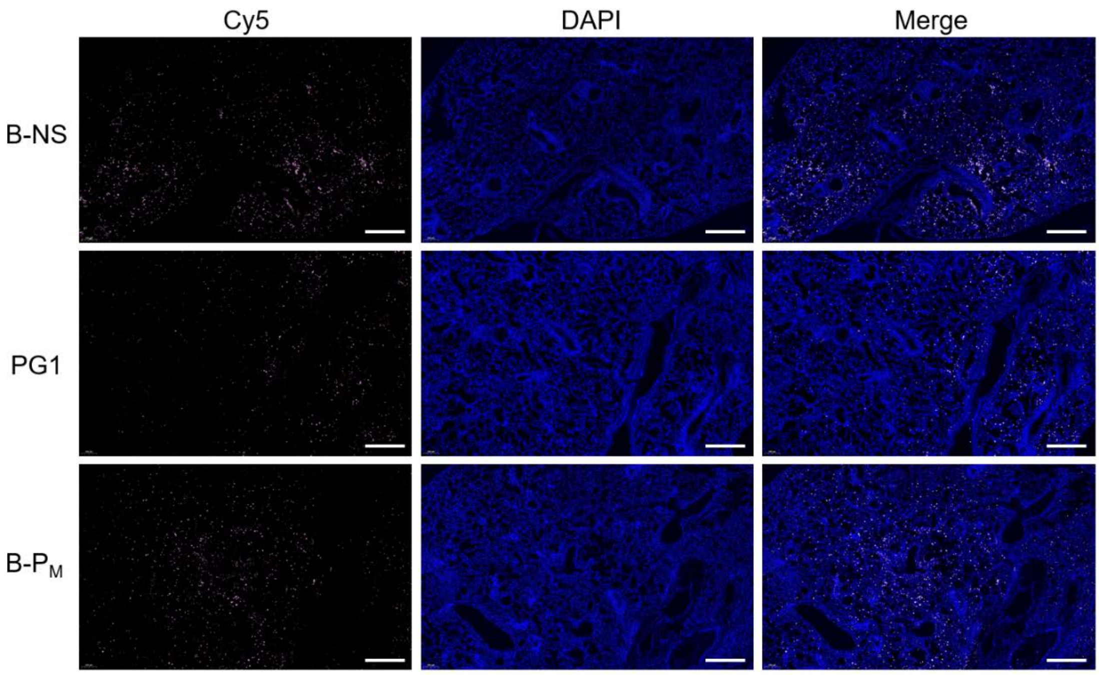

Figure S16. Biodistribution of B-NS, PG1, and B-P$_M$ in the lungs of experimental mice. Distribution of Cy5-labeled B-NS, PG1, and B-P$_M$ in lungs of model mice at 1 d after treatment. Scale bars: 500 µm.

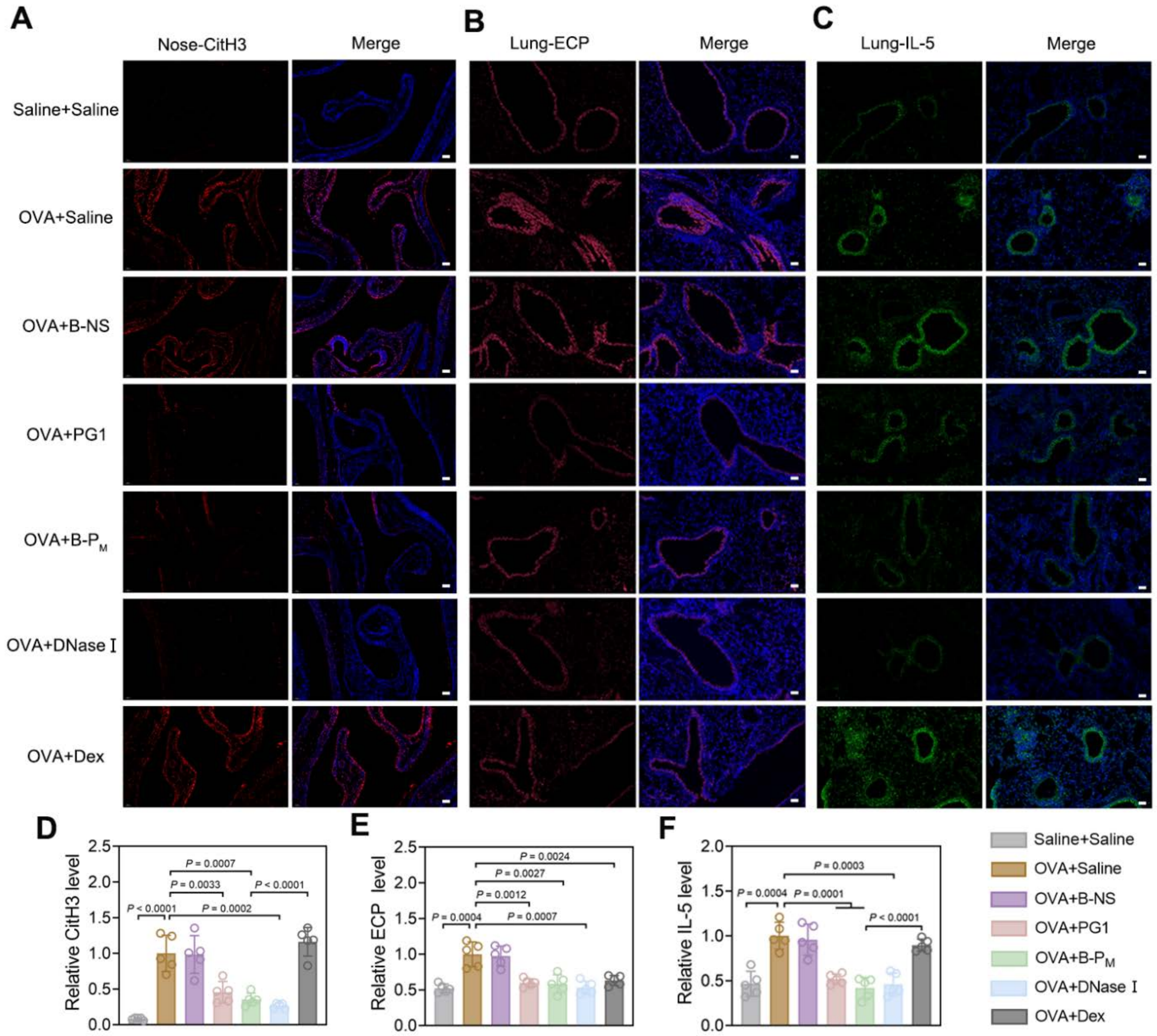

Figure S17. Anti-inflammation effect of B-P$_M$ for nasal mucosa and lungs. (**A**) Representative CitH3 immunostaining images of nasal mucosa of mice in each group. (**B–C**) Representative images of (**B**) ECP and (**C**) IL-5 immunostaining in the lungs of mice in each group. Scale bars: 50 µm. (**D–F**) Quantitative analysis of (**D**) CitH3 in (**A**), (**E**) ECP in (**B**), and (**F**) IL-5 in (**C**). Data are presented as mean ± SD (n = 5, Student's t-test, two-tailed).

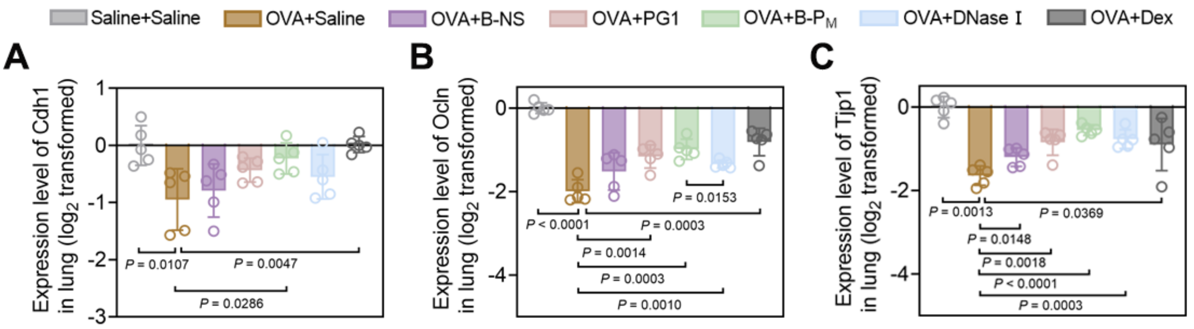

Figure S18. Therapeutic effect of B-P$_M$ on lung epithelial injury. (**A–C**) The relative expression levels of (**A**) Cdh1, (**B**) Ocln, and (**C**) Tjp1 in the lungs of experimental mice in each group. Data are presented as mean ± SD (n = 5, Student's t-test, two-tailed).

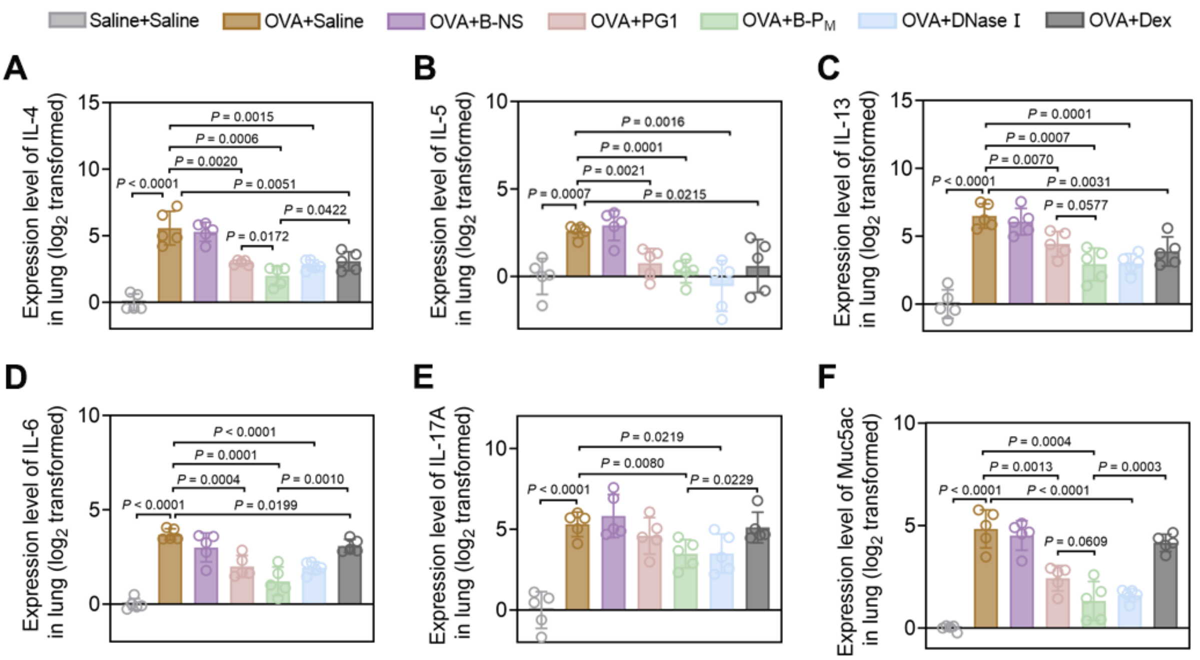

Figure S19. Alleviation of lung inflammation by B-P$_M$ *in vivo* (qRT-PCR). (**A–F**) The expression levels of (**A**) IL-4, (**B**) IL-5, (**C**) IL-13, (**D**) IL-6, (**E**) IL-17A, and (**F**) Muc5ac in the lungs of mice in each group. Data are presented as mean ± SD (n = 5, Student's t-test, two-tailed).

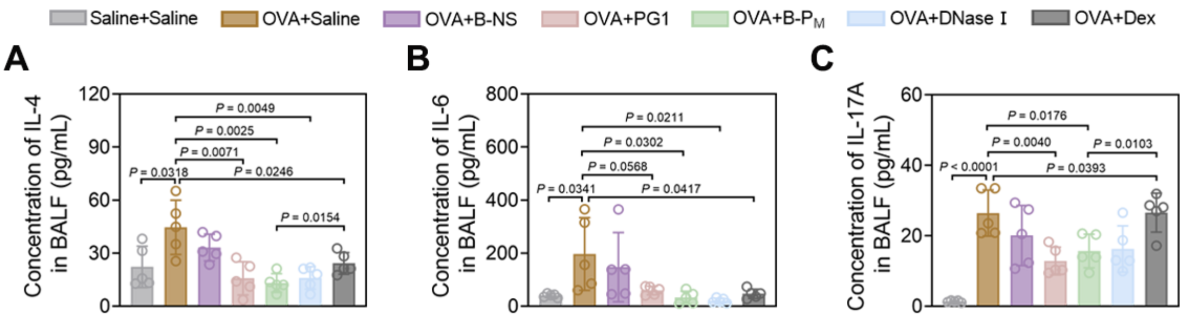

Figure S20. Alleviation of lung inflammation by B-P$_M$ *in vivo* (ELISA). (**A–C**) Protein concentrations of (**A**) IL-4, (**B**) IL-6, and (**C**) IL-17A in BALF of mice in each group. Data are presented as mean ± SD (n = 5, Student's t-test, two-tailed).

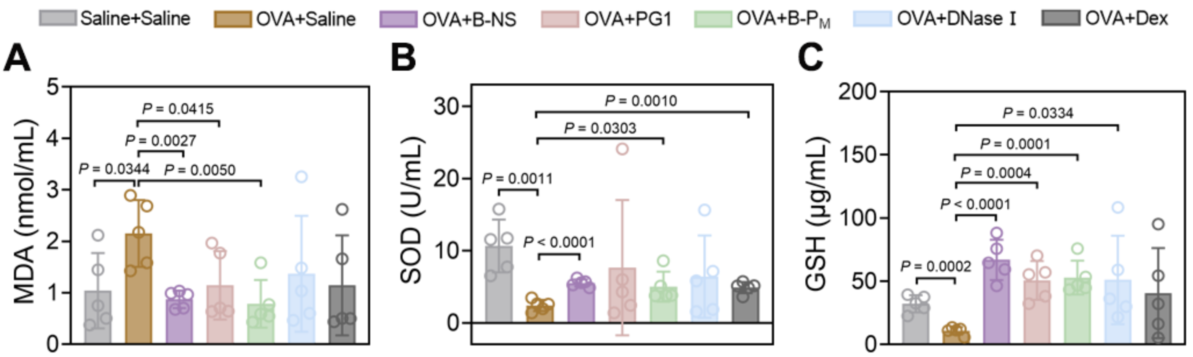

Figure S21. Regulation of redox levels by B-P$_M$ *in vivo*. (**A**) MDA, (**B**) SOD, and (**C**) GSH levels in BALF of mice in each group. Data are presented as mean ± SD (n = 5, Student's t-test, two-tailed).

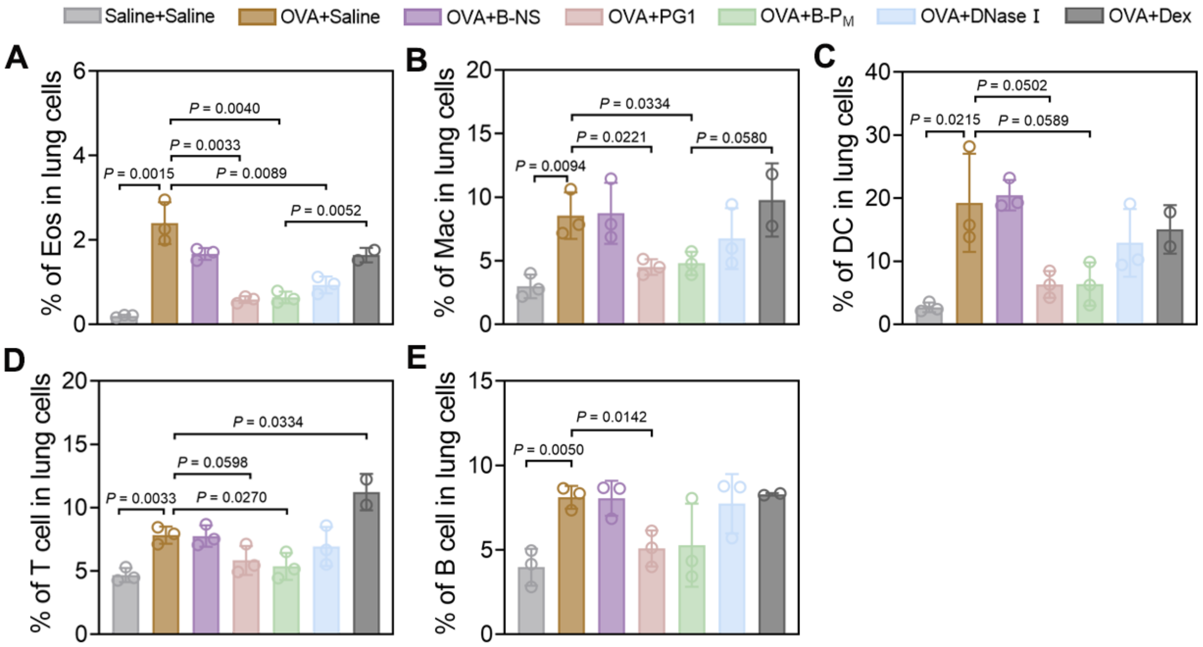

Figure S22. Quantification of the proportion of immune cells in the lungs of experimental mice based on t-SNE analysis. (**A–E**) Percentage of (**A**) eosinophils, (**B**) macrophages, (**C**) dendritic cells, (**D**) T cells, and (**E**) B cells in total lung cells of mice in each group. Data are presented as mean ± SD (Student's t-test, two-tailed).

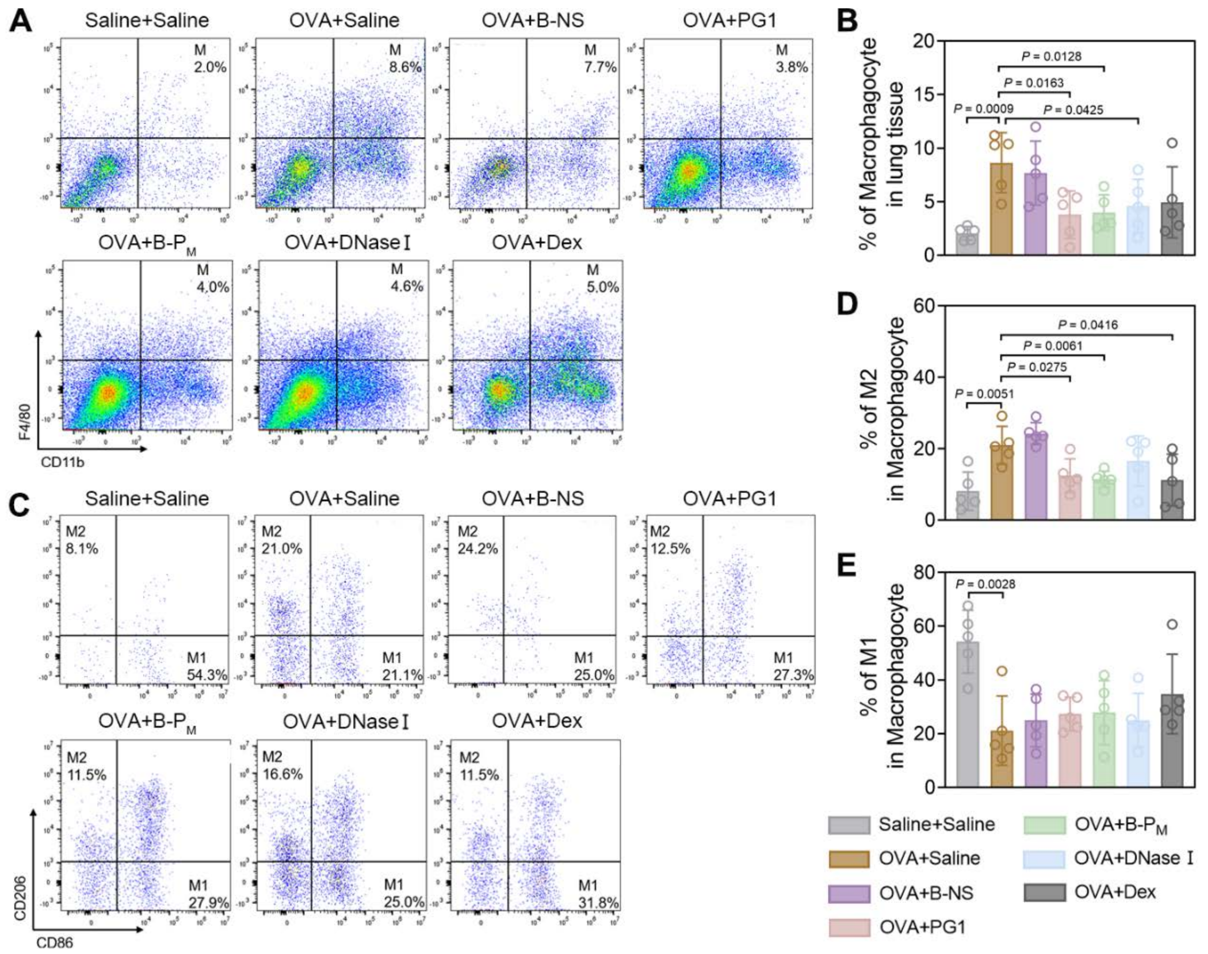

Figure S23. Analysis of macrophage phenotype in lungs of experimental mice after B-P$_M$ treatment. (**A**) Flow cytometric plots of Mac in the lungs of mice in each group. (**B**) Quantitative analysis of Mac in (**A**). (**C**) Flow cytometric plots of M1 and M2 in the lungs of mice in each group. (**D–E**) Quantitative analysis of (**D**) M2 and (**E**) M1 in (**C**). Data are presented as mean ± SD (n = 5, Student's t-test, two-tailed).

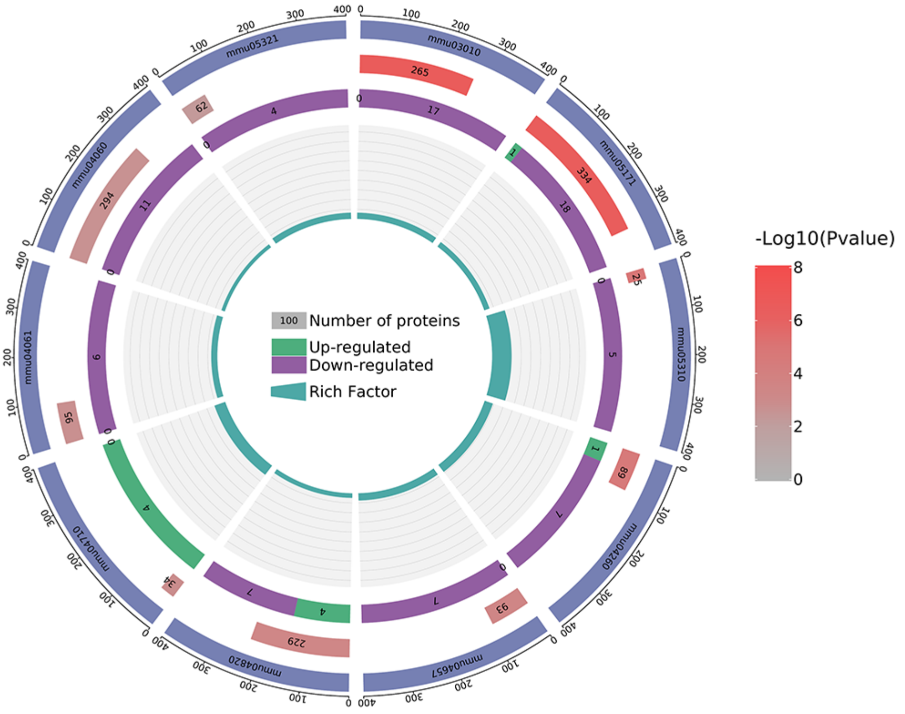

Figure S24. Reactome circle of KEGG enrichment entries of DEGs after B-P$_M$ treatment. Visualization of enrichment count number, up-/down-regulated gene number, enrichment factor, and significance degree of KEGG enrichment items.

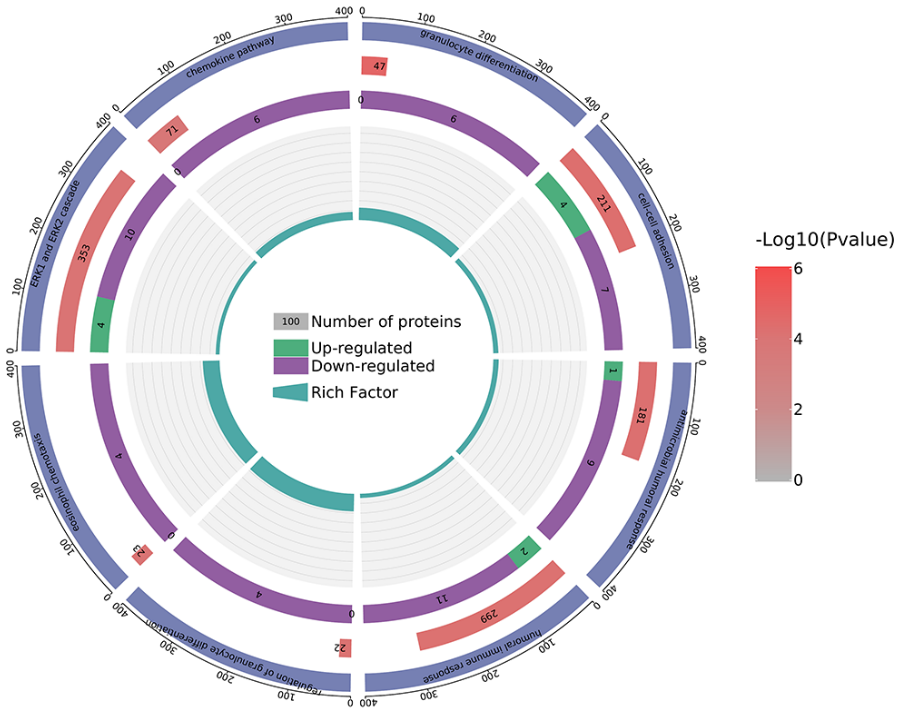

Figure S25. Reactome circle of GO enrichment entries of DEGs after B-P$_M$ treatment. Visualization of enrichment count number, up-/down-regulated gene number, enrichment factor, and significance degree of part of inflammation-related GO enrichment items.

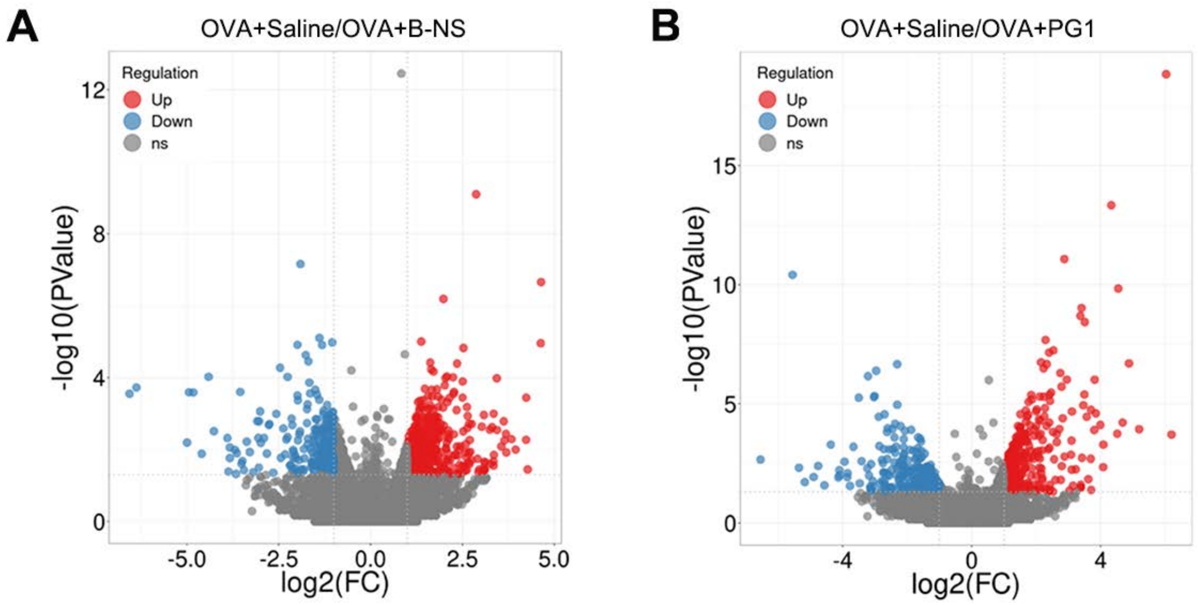

Figure S26. Volcano map of DEGs. (**A**-**B**) Volcano map between (**A**) inflammatory group and B-NS-treated group, (**B**) inflammatory group and PG1-treated group (Threshold: |log2(FC)|≥1.0, p≤0.05).

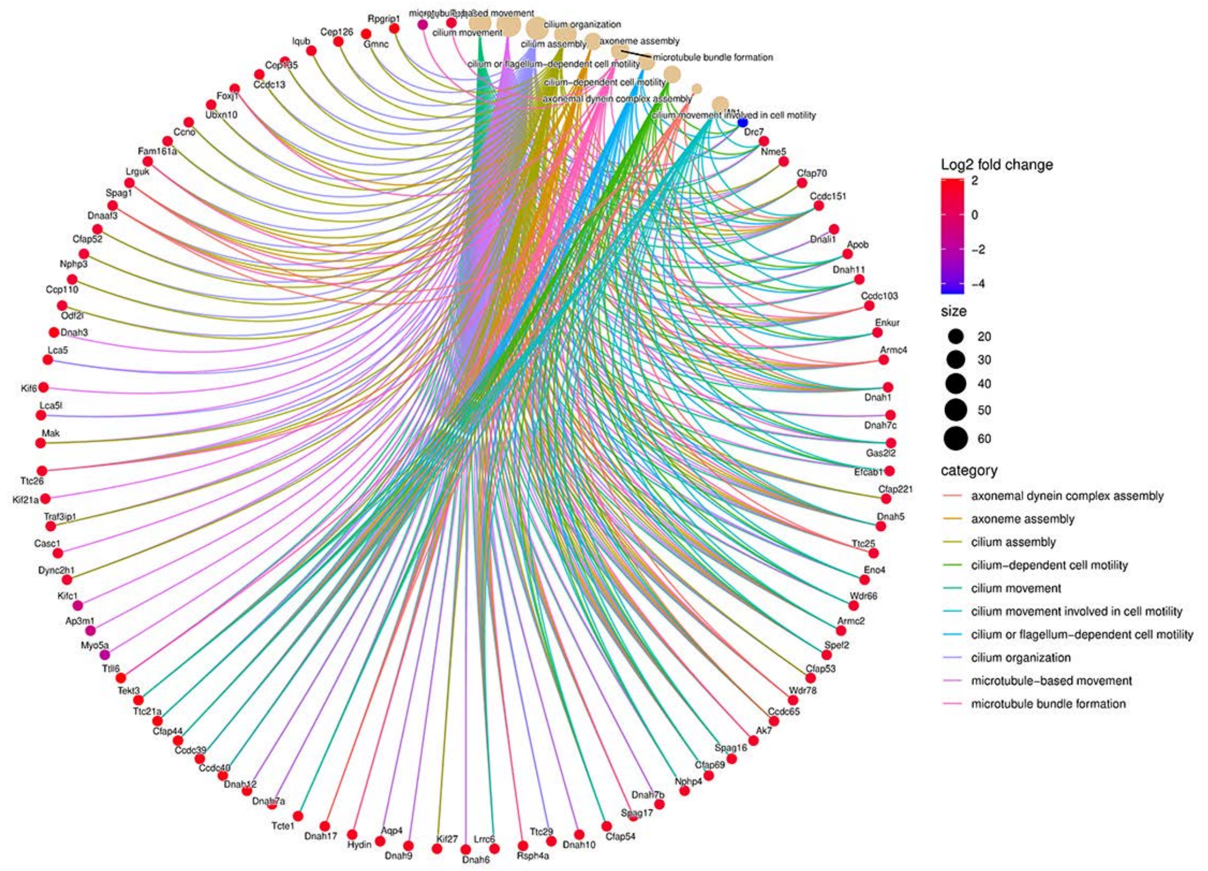

Figure S27. The chord diagram of GO enrichment entries of DEGs after B-NS treatment. The upper right circle represents the enriched GO entries, the remaining circle represents the differential proteins, and the color represents the differential multiples, red represents the up-regulated differential proteins, and blue represents the down-regulated differential proteins.

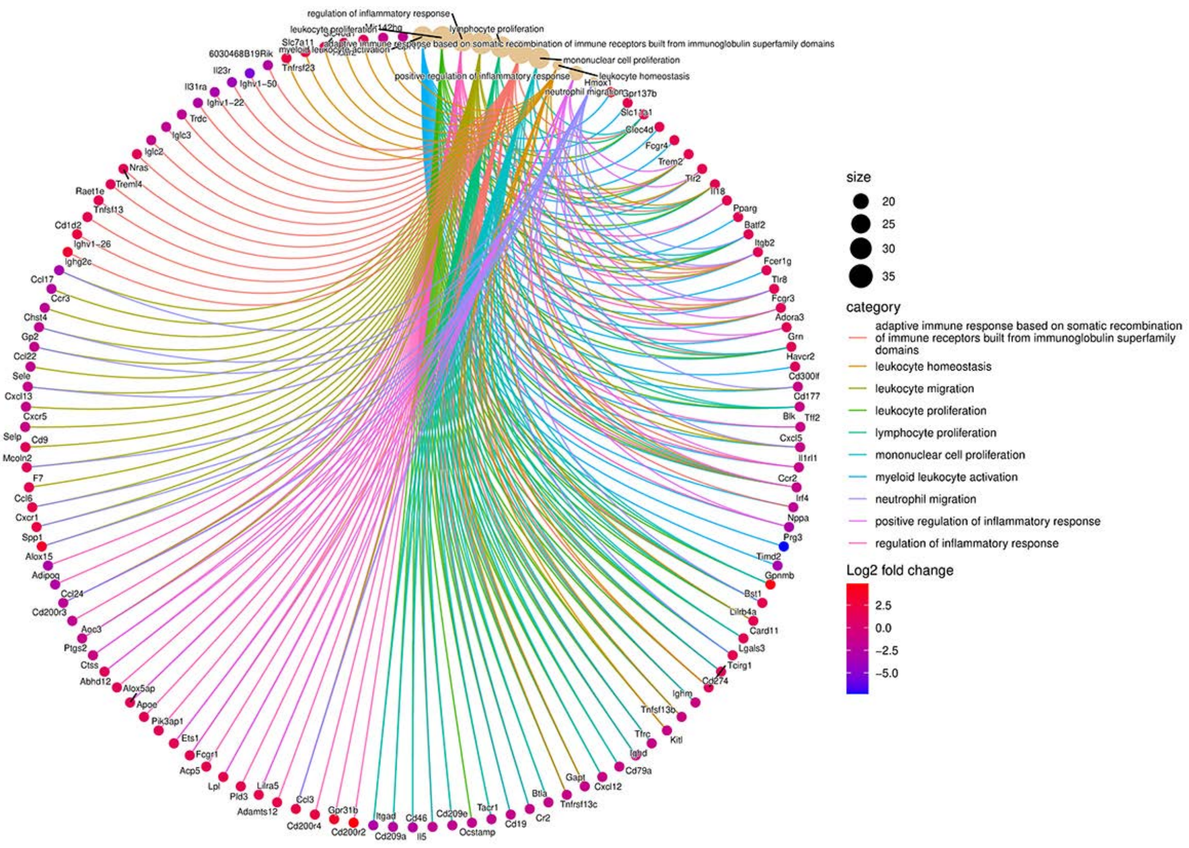

Figure S28. The chord diagram of GO enrichment entries of DEGs after PG1 treatment. The upper right circle represents the enriched GO entries, the remaining circle represents the differential proteins, and the color represents the differential multiples, red represents the up-regulated differential proteins, and blue represents the down-regulated differential proteins.

References

[1] D. Saroj Kumar, B. Amita, K. Aadithya, J. Kabeer, Aqueous dispersions of few-layer-thick chemically modified magnesium diboride nanosheets by ultrasonication assisted exfoliation, Sci Rep, 5 (2015).
